# Epigenomic analysis reveals a unique DNA methylation program of metastasis-competent circulating tumor cells in colorectal cancer

**DOI:** 10.1038/s41598-023-42037-w

**Published:** 2023-09-16

**Authors:** Aida Bao-Caamano, Nicolás Costa-Fraga, Laure Cayrefourcq, María Amalia Jácome, Aitor Rodriguez-Casanova, Laura Muinelo-Romay, Rafael López-López, Catherine Alix-Panabières, Angel Díaz-Lagares

**Affiliations:** 1 Epigenomics Unit, Cancer Epigenomics, Translational Medical Oncology Group (ONCOMET), Health Research Institute of Santiago de Compostela (IDIS), University Clinical Hospital of Santiago (CHUS/SERGAS), 15706 Santiago de Compostela, Spain; 2 Universidade de Santiago de Compostela (USC), 15782 Santiago de Compostela, Spain; 3 Galician Precision Oncology Research Group (ONCOGAL), Medicine and Dentistry School, Universidade de Santiago de Compostela (USC), Santiago de Compostela, Spain; 4 Laboratory of Rare Human Circulating Cells, University Medical Center of Montpellier, IURC, 641, Avenue du Doyen Gaston Giraud, 34093 Montpellier Cedex 5, France; 5 CREEC, MIVEGEC, University of Montpellier, CNRS, IRD, Montpellier, France; 6 Department of Mathematics, MODES Group, CITIC, Faculty of Science, Universidade da Coruña, A Coruña, Spain; 7 Roche-Chus Joint Unit, Translational Medical Oncology Group (ONCOMET), Health Research Institute of Santiago (IDIS), 15706 Santiago de Compostela, Spain; 8 Liquid Biopsy Analysis Unit, Translational Medical Oncology Group (ONCOMET), Health Research Institute of Santiago de Compostela (IDIS), 15706 Santiago de Compostela, Spain; 9 Centro de Investigación Biomédica en Red Cáncer (CIBERONC), ISCIII, 28029 Madrid, Spain; 10 Translational Medical Oncology Group (ONCOMET), Health Research Institute of Santiago de Compostela (IDIS), University Clinical Hospital of Santiago (CHUS/SERGAS), 15706 Santiago de Compostela, Spain; 11 European Liquid Biopsy Society (ELBS), Hamburg, Germany; 12 Department of Clinical Analysis, University Hospital Complex of Santiago de Compostela (CHUS), 15706 Santiago de Compostela, Spain

**Keywords:** Metastasis, Cancer

## Abstract

Circulating tumor cells (CTCs) and epigenetic alterations are involved in the development of metastasis from solid tumors, such as colorectal cancer (CRC). The aim of this study was to characterize the DNA methylation profile of metastasis-competent CTCs in CRC. The DNA methylome of the human CRC-derived cell line CTC-MCC-41 was analyzed and compared with primary (HT29, Caco2, HCT116, RKO) and metastatic (SW620 and COLO205) CRC cells. The association between methylation and the transcriptional profile of CTC-MCC-41 was also evaluated. Differentially methylated CpGs were validated with pyrosequencing and qMSP. Compared to primary and metastatic CRC cells, the methylation profile of CTC-MCC-41 was globally different and characterized by a slight predominance of hypomethylated CpGs mainly distributed in CpG-poor regions. Promoter CpG islands and shore regions of CTC-MCC-41 displayed a unique methylation profile that was associated with the transcriptional program and relevant cancer pathways, mainly Wnt signaling. The epigenetic regulation of relevant genes in CTC-MCC-41 was validated. This study provides new insights into the epigenomic landscape of metastasis-competent CTCs, revealing biological information for metastasis development, as well as new potential biomarkers and therapeutic targets for CRC patients.

## Introduction

Colorectal cancer (CRC) is one of the main causes of cancer-related deaths in the world^1^, with 0.9 million deaths in 2020^2^. The 5‐year survival rate ranges from 90 to 14% depending on whether they are diagnosed at early or advanced stages^3^. The mortality of this disease is highly associated with the development of the metastasis cascade, in which circulating tumor cells (CTCs) serve a fundamental causal role^4,5^. CTCs are tumor cells that spread from primary tumors (or metastatic sites) and can colonize distant tissues to form metastases^5,6^. The study of CTCs in recent years has provided valuable information about cancer metastasis, leading to the development of clinical applications. In this sense, the enumeration of CTCs in the peripheral blood of metastatic CRC patients provides prognostic information about the outcome of this disease^7^. Although relevant advances in understanding of the molecular properties of CTCs have been obtained in recent years, the molecular characterization of CTCs continues to be very challenging due to the low presence of these cells in the peripheral blood of cancer patients^5^. Therefore, it is important to take advantage of CTC-derived cell line models to increase our knowledge about the metastatic process^8^.

Recently, Cayrefourcq et al*.* established the first human CTC-derived cell line of CRC, designated CTC-MCC-41. This cell line was isolated from the peripheral blood of a nonresectable metastatic CRC patient before starting first-line chemotherapy. CTC-MCC-41 cells are metastasis-competent cells with a stable intermediate epithelial-mesenchymal phenotype and stem cell-like properties^9^. This cell line has been characterized at the transcriptional level, revealing the differential expression of genes involved in relevant cancer-related pathways, such as energy metabolism and DNA repair^10^.

DNA methylation is an epigenetic mechanism that regulates gene expression. This epigenetic modification consists of the incorporation of a methyl group (CH_3_) into the 5′ carbon of cytosines in cytosine-phosphate-guanine (CpG) dinucleotides to produce 5-methylcytosine (5mC)^11^. Cancer cells are characterized by an aberrant DNA methylation profile, which may be different between primary and metastatic tumors^12,13^. Recently, it has been described that colorectal CTCs may undergo aberrant methylation in some particular genes^14^. However, the DNA methylome of colorectal CTCs with the ability to initiate and support metastasis formation is still unexplored. In this context, it is important to characterize the DNA methylation pattern of this subset of CTCs to better understand the metastatic process and obtain new clinical applications for cancer patients.

Therefore, in this study, we analyzed the DNA methylome of the metastasis-competent CTC-MCC-41 cell line in comparison with primary (HT29, Caco2, HCT116, RKO) and metastatic (SW620 and COLO205) CRC cell lines. This epigenomic analysis revealed that metastasis-competent cancer CTCs displayed a distinct methylation program with respect to primary and metastatic tumor cells which is able to regulate the transcriptional profile of CTCs. This analysis enabled the identification of aberrantly DNA methylated genes and pathways, providing relevant biological information in the context of metastasis and discovering potential biomarkers and therapeutic targets of CTCs with clinical implications for cancer patients.

## Materials and methods

### Cancer cell lines and treatments

The characteristics of all the human CRC cell lines used in this study are indicated in Supplementary Table 1. The cell line CTC-MCC-41 (RRID:CVCL_0I26) was recently stablished and characterized by coauthors of this work^9,10,15^. This cell line was cultured using Corning Ultra-Low attachment cell culture flasks (Merck) and RPMI-1640 medium (Merck) supplemented with 10% fetal bovine serum (FBS) (Merck), 1% penicillin/streptomycin solution (Gibco, Thermo Fisher Scientific), 1% l-glutamine (Merck), 1% insulin-transferrin-selenium (ITS-G) (Gibco, Thermo Fisher Scientific) basic human fibroblast growth factor (bFGF) (Miltenyi Biotec) at a final concentration of 10 ng/mL and human epidermal growth factor (hEGF) (Miltenyi Biotec) at a final concentration of 20 ng/mL. The primary tumor cell lines HT29 (RRID:CVCL_0320), Caco2 (RRID:CVCL_0025), HCT116 (RRID:CVCL_0291), and RKO (RRID:CVCL_0504); and the metastatic cell lines COLO205 (RRID:CVCL_0218) and SW620 (RRID:CVCL_0547) used in this study were all purchased from the American Type Culture Collection (ATCC) (Rockville, MD). The cell lines from ATCC were cultured according to its recommendations with the following media: RKO and Caco2 with EMEM, HT29 and HCT116 with McCoy's 5A, COLO205 with RPMI-1640, and SW620 with Leibovitz's L-15. All media were supplemented with 1% penicillin/streptomycin (Gibco, Thermo Fisher Scientific) and 10% FBS (Merck), except for Caco2 (HTB-37, ATCC®), for which 20% FBS was used according to ATCC culture guides and previous publications^16^. All the cells used in this study were grown at 37 °C with 5% CO_2_, and all experiments were performed with mycoplasma-free cells. To promote DNA demethylation, the CTC-MCC-41 cell line was treated with 5-aza-2′-deoxycytidine (AZA). Briefly, 2.5 × 10^6^ cells were plated in Corning™ Ultra-Low attachment cell culture flasks (Merck) and treated with 5 μM AZA for 72 h. DMSO (Merck) was used as a control.

### Isolation of DNA and RNA from cell lines

Total genomic DNA was isolated from cell lines using a standard high salt method based on SDS/Proteinase K. The isolated DNA was treated with RNAse A (Qiagen) following the manufacturer's recommendations and stored at − 80 °C until analysis. All DNA samples were quantified with the Qubit 4 fluorometric method (Invitrogen) using the Qubit dsDNA BR (Broad Range) Assay Kit (Invitrogen). Total RNA was isolated from cell lines using TRIzol (Invitrogen) according to the manufacturer's protocol, quantified using a NanoDrop One spectrophotometer (Thermo Scientific) and stored at − 80 °C until analysis.

### Genome-wide DNA methylation analysis by EPIC arrays

Total genomic DNA (500 ng) of 3 passages of the cell lines CTC-MCC-41 (P12, P13 and P14), HT29 (P2, P3 and P4), and COLO205 (P2, P3 and P4) was converted by sodium bisulfite using the EZ DNA Methylation kit (Zymo Research). In addition, 500 ng of total genomic DNA from one passage of the cell lines Caco2, HCT116, RKO, and SW620 was also converted by sodium bisulfite with the EZ DNA Methylation kit (Zymo Research). Following the manufacturer's protocol, the bisulfite-converted DNA was hybridized using the Infinium MethylationEPIC array (EPIC), which cover over 850,000 CpG sites along the human genome. Whole-genome amplification and hybridization were performed, followed by single-base extension and analysis on a HiScan (Illumina) to assess the cytosine methylation states. Image intensities were extracted using GenomeStudio (V2011.1) Methylation Module (1.9.0) software from Illumina. Data quality control was assessed with GenomeStudio and BeadArray Controls Reporter (Illumina) based on the internal control probes present on the array. The methylation score of each CpG from samples that passed this quality control was represented as the β-value and previously normalized for color bias and background level adjustment and quantile normalization across arrays. β-values were obtained as the ratio of the fluorescent signal of the methylated (M) probe relative to the sum of the M and unmethylated (U) probes (β = M/(M + U)). The β-values range from 0 (no methylation) to 1 (completely methylated). Probes and sample filtering involved a two-step process for removing SNPs and unreliable β-values with a high detection p value > 0.01. After this filtering step, the remaining CpGs were considered valid for the study. Unsupervised hierarchical clustering heatmaps with the ComplexHeatmap package (2.10.0)^17^, scatter plots and principal component analysis (PCA) of β-values were carried out using the R environment. Gene Ontology (GO) enrichment analysis of biological and Panther pathways for the methylation profiles was evaluated using GENECODIS.

### Locus-specific DNA methylation analysis by pyrosequencing and qMSP

Total genomic DNA (500 ng) of 3 passages of the cell lines CTC-MCC-41 (P12, P13 and P14) and HT29 (P2, P3 and P4) was converted by sodium bisulfite with the EZ DNA Methylation kit (Zymo Research) following the manufacturer’s recommendations. For pyrosequencing, primer sequences (Supplementary Table 2) were designed with PyroMark Assay Design 2.0 (Qiagen). Standard PCRs were carried out with ~ 10 ng of bisulfite-converted genomic DNA. PCR products were observed with 2% agarose gels before pyrosequencing. A PyroMark Q24 Vacuum Workstation was used for the immobilization and preparation of PCR products. Pyrosequencing reactions were performed using a PyroMark Gold Q24 Reagent Kit (Qiagen, Germany) following the manufacturer’s instructions. Methylation values were obtained using PyroMark Q24 Software 2.0 (Qiagen). For quantitative methylation-specific PCR (qMSP), primer sequences (Supplementary Table 3) were designed using Primer3 (v. 0.4.0). The DNA methylation levels for qMSP were determined in a StepOne Plus system (Applied Biosystems). Each reaction contained 2 µL of bisulfite-converted DNA as a template, 10 µL *Power* SYBR™ Green PCR Master Mix (Thermo Fisher) and 0.3 µL of each forward and reverse 10 µM primer in a total volume of 20 µL. The DNA methylation level determined by qMSP was expressed as a percentage of methylation (%) according to the following formula^18^: Methylation (%) = 100/[1 + 2^(CT_CG_–CT_TG_)], where CT_CG_ and CT_TG_ represent the threshold cycle (CT) of the methylation and unmethylation status, respectively. The methylation assays were conducted in triplicate using the Human Methylated & Nonmethylated DNA set (Zymo Research) as positive and negative controls. Water was used as a no-template control.

### Gene expression data obtained from microarray analysis

The gene expression data from the cell lines CTC-MCC-41 and HT29 analyzed with microarray methodology were obtained from a previous work by Alix-Panabières et al*.*^10^. Briefly, in this study, the transcriptomes (total RNA) of CTC-MCC-41 and HT29 cells were analyzed using Human Genome U133 Plus 2.0 GeneChip arrays (Affymetrix).

### Gene expression analysis by qRT‒PCR

For quantitative RT‒PCR (qRT‒PCR), the isolated RNA was first treated with DNase I using the Turbo DNA-free Kit (Invitrogen) according to the manufacturer's recommendations. Next, 1–2 μg of RNA was retrotranscribed using the SuperScript First-Strand Synthesis System for RT‒PCR (Invitrogen) according to the manufacturer's recommendations. Reactions were performed in triplicate on a StepOne Plus system (Applied Biosystems) using 25–200 ng cDNA, 10 μL *Power* SYBR Green PCR Master Mix (Applied Biosystems) and 0.3 μL of the 10 μM specific primers in a final volume of 20 μL. The results were normalized to the expression level of β_2_-microglobulin (endogenous control) in each sample. The primers used for qRT‒PCR analysis were previously described^10^ and are indicated in Supplementary Table 4.

### Statistical analysis

For genome-wide DNA methylation analysis, differentially methylated CpGs (DMCpGs) were determined using empirical Bayes methodology. P values were corrected for multiple testing (false discovery rate, FDR) using the Benjamini‒Hochberg method, and a threshold of p value < 0.05 was selected for significance. For the validation assay of DNA methylation levels and for the evaluation of expression differences after AZA treatment, the Kolmogorov‒Smirnov test was first used to evaluate the normality of the distribution, and the Mann‒Whitney *U* test or Student´s t test was used accordingly. GraphPad Prism 6.0 software and the R statistical environment (version 4.2.0) were used for statistical analysis. All expressed p values were calculated with two-tailed tests and were considered significant when the p value < 0.05.

## Results

### CTC-MCC-41 cells show a globally different DNA methylation profile than primary HT29 tumor cells

**Figure 1:**
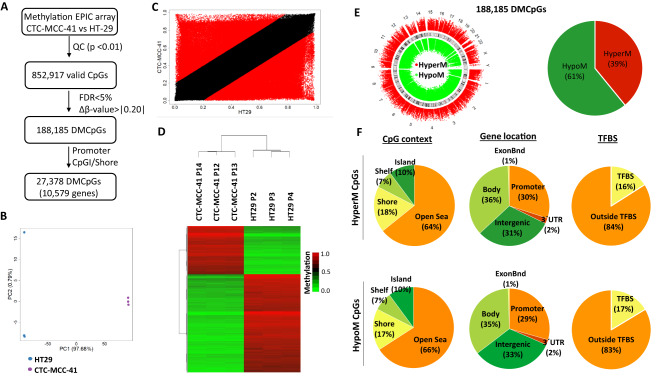
Genome-wide DNA methylation analysis of CTC-MCC-41 with respect to HT29 primary tumor cells. (**A**) Schematic flowchart used to identify significant differentially methylated CpGs in CTC-MCC-41 compared to HT29. (**B**) Principal component analysis (PCA) of DNA methylation data obtained in CTC-MCC-41 and HT29 cells. (**C**) Scatter plot representing the mean normalized levels of DNA methylation (β-values) in CTC-MCC-41 and HT29 cells. Dots in red show significantly differentially methylated CpGs. (**D**) Hierarchical clustering heatmap of the 10,000 most differentially methylated CpGs (FDR adjusted p value < 0.05) between CTC-MCC-41 and HT29. Heatmap shows three different passages (P) of CTC-MCC-41 (P12, P13 and P14) and HT29 (P2, P3 and P4). (**E**, **F**) Description of the 188,185 differentially methylated CpGs (DMCpGs) found in CTC-MCC-41 with respect to HT29 according to (**E**) chromosome location and methylation status and (**F**) CpG context, gene location and transcription factor-binding sites (TFBS). *QC* quality control, *FDR* false discovery rate, *CpGI* CpG island, *HypoM* hypomethylated, *HyperM* hypermethylated.

The DNA methylome of CTC-MCC-41 was evaluated with respect to the primary CRC cell line HT29 using EPIC arrays. After quality control, we obtained 852,917 valid CpGs for the analysis (Fig. 1A). Principal component analysis (PCA) of these CpGs revealed a different methylation profile between CTC-MCC-41 and HT29 (Fig. 1B), with both cell types classified into different groups. In agreement with this, CTC-MCC-41 also showed a very different methylation profile with respect to HT29 (R^2^ = 0.68) (Fig. 1C). In particular, we were able to identify 188,185 significant differentially methylated CpGs (DMCpGs) (FDR adjusted p value < 0.05) with a difference of methylation (Δβ-value) greater than 0.20 (Δβ-value > |0.20|) between CTC-MCC-41 and HT29 cancer cells. Notably, a hierarchical clustering analysis revealed a DNA methylation profile able to clearly differentiate CTC-MCC-41 from HT29 cancer cells (Fig. 1D). Supplementary Table 5 shows the top 50 DMCpGs found between CTC-MCC-41 and HT29.

The 188,185 DMCpGs previously identified showed a wide distribution throughout all the chromosomes of the genome, showing a higher number of hypomethylated CpGs (114,621 CpGs; 61% of all DMCpGs) than hypermethylated CpGs (73,564 CpGs; 39% of all CpGs) in CTC-MCC-41 respect to HT29 cancer cells (Fig. 1E). Interestingly, the hypo- and hypermethylated CpGs of CTC-MCC-41 showed a similar distribution according to their CpG context and gene region location (Fig. 1F). In particular, most of these DMCpGs were mainly located in regions with low CpG density (open sea), whereas the other CpGs were distributed in regions with a higher enrichment of CpGs, such as CpG islands (CpGIs) and shore regions. Regarding the gene region, both the hypo- and hypermethylated CpGs were fairly homogeneously distributed throughout promoters, gene bodies and intergenic regions. In addition, although most of the CpGs located at transcription factor-binding sites (TFBS) did not show methylation changes, some of these CpGs were differentially methylated. These methylation differences corresponded with a higher number of hypomethylated CpGs located at TFBS (19,651 CpGs; 17% of all the DMCpGs at TFBS) of CTC-MCC-41 cells than hypermethylated CpGs (11,762 CpGs; 16% of all the DMCpGs at TFBS). On the other hand, enhancer regions also showed DMCpGs, but these differences corresponded with a small proportion of the CpGs (< 4%) located at these regulatory regions (Supplementary Fig. 1).

### CTC-MCC-41 cells have a different DNA methylation profile at CpGIs and shore regions of gene promoters than primary HT29 tumor cells

**Figure 2:**
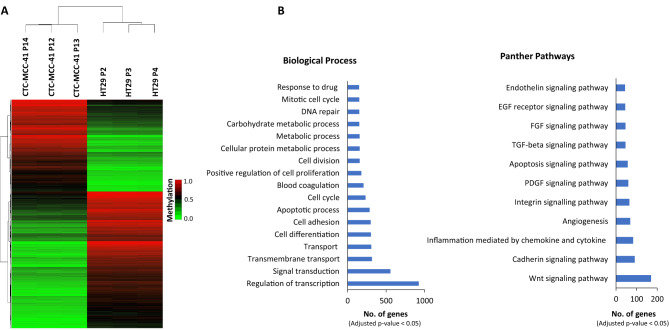
DNA methylation profiles of CpGIs and shore regions of gene promoters in CTC-MCC-41 compared to HT29 primary tumor cells. (**A**) Hierarchical clustering heatmap of the 10,000 most differentially methylated CpGs (FDR adjusted p value < 0.05) in CTC-MCC-41 with respect to HT29 and located at CpGIs and shore regions of gene promoters. Heatmap shows three different passages (P) of CTC-MCC-41 (P12, P13 and P14) and HT29 (P2, P3 and P4). (**B**) Gene Ontology (GO) analysis representing some of the most cancer-relevant biological processes and Panther pathways based on the 10,000 most differentially methylated CpGs of CTC-MCC-41 compared to HT29 and located at CpGIs and shore regions of gene promoters.

The epigenetic deregulation of CpGIs and shores of gene promoters has been described as a very relevant feature that occurs in cancer^19,20^. Therefore, we decided to focus our analysis on CpGs located in these particular regions. Thus, we identified 27,378 CpGs located in CpGIs or shore regions of gene promoters (corresponding to 10,579 genes) differentially methylated between CTC-MCC-41 and HT29 (Fig. 1A). Importantly, using hierarchical clustering analysis, we identified a methylation profile at these CpG-rich promoter regions that was able to clearly differentiate CTC-MCC-41 from HT29 cancer cells (Fig. 2A). The top 50 DMCpGs from CpGIs or shore regions of gene promoters are represented in Supplementary Table 6. In addition, we carried out a Gene Ontology (GO) and Panther pathway analysis with the differentially methylated genes from these CpG-rich promoters, revealing an enrichment of relevant biological processes (regulation of transcription, cell adhesion and differentiation, apoptosis, cell cycle and proliferation, metabolic processes and DNA repair, among others) and pathways (such as Wnt signaling, cadherin pathway, inflammation, angiogenesis and integrin signaling) related to cancer development and metastasis (Fig. 2B and Supplementary Fig. 2). The top 10 differentially methylated genes related to each biological process and pathway are indicated in Supplementary Table 7–8.

### CTC-MCC-41 cells exhibit a globally different DNA methylation profile than metastatic COLO205 tumor cells

**Figure 3:**
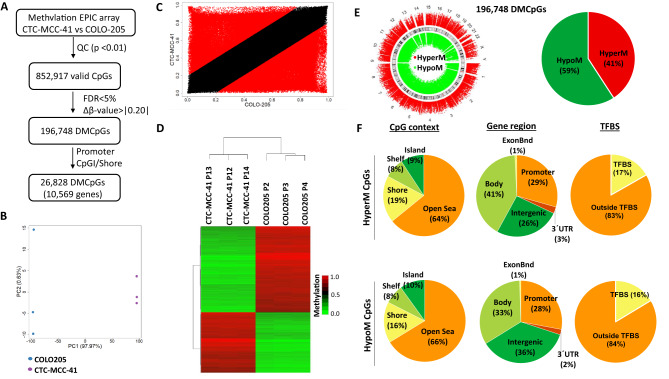
Genome-wide DNA methylation analysis of CTC-MCC-41 with respect to COLO205 metastatic tumor cells. (**A**) Schematic flowchart used to identify significantly differentially methylated CpGs in CTC-MCC-41 compared to COLO205. (**B**) Principal component analysis (PCA) of DNA methylation data obtained for CTC-MCC-41 and COLO205 cells. (**C**) Scatter plot representing the mean normalized levels of DNA methylation (β-values) in CTC-MCC-41 and COLO205 cells. Dots in red show significantly differentially methylated CpGs. (**D**) Hierarchical clustering heatmap of the 10,000 most differentially methylated CpGs (FDR adjusted p value < 0.05) between CTC-MCC-41 and COLO205. Heatmap shows three different passages (P) of CTC-MCC-41 (P12, P13 and P14) and COLO205 (P2, P3 and P4). (**E**, **F**) Description of the 188,185 differentially methylated CpGs (DMCpGs) found in CTC-MCC-41 with respect to COLO205 according to (**E**) chromosome location and methylation status and (**F**) CpG context, gene location and transcription factor-binding sites (TFBS). *QC* quality control, *FDR* false discovery rate, *CpGI* CpG island, *HypoM* hypomethylated, *HyperM* hypermethylated.

Similar to our previous analysis, the DNA methylome of CTC-MCC-41 was also evaluated with respect to the metastatic CRC cell COLO205 with EPIC arrays, allowing the analysis of 852,917 valid CpGs (Fig. 3A). The evaluation of the methylation levels of these CpGs using PCA and scatter plot (R^2^ = 0.66) showed that CTC-MCC-41 and COLO205 have different global methylation profiles (Fig. 3B,C). Among the CpGs analyzed, we identified 196,748 significant DMCpGs (FDR adjusted p value < 0.05; Δβ-value > |0.20|) between CTC-MCC-41 and COLO205. Importantly, a hierarchical clustering analysis of these DMCpGs identified a methylation profile that clearly differentiates the CTC-MCC-41 from the COLO205 cancer cells (Fig. 3D). The top 50 DMCpGs in CTC-MCC-41 with respect to COLO205 are represented in Supplementary Table 9.

The analysis of the 196,748 DMCpGs previously obtained showed that methylation differences are distributed throughout the entire genome. Similar to our previous comparison between CTC-MCC-41 and HT29, most of the DMCpGs found in CTC-MCC-41 were hypomethylated with respect to COLO205 (116,431 CpGs; 59% of all DMCpGs), while the rest (80,317 CpGs; 41% of all DMCpGs) were hypermethylated (Fig. 3E). These DMCpGs showed a similar distribution according to their CpG context and gene region location (Fig. 3F), with most of the CpGs located in regions with low CpG density (open sea). In addition, the distribution of these CpGs was fairly similar between promoters, gene bodies and intergenic regions (Fig. 3F). We also found DMCpGs located at TFBS, but these CpGs represented a small fraction of the total CpGs from the TFBS. The methylation differences found at TFBS corresponded with a higher number of hypomethylated CpGs (19,068 CpGs; 16% of the DMCpGs at TFBS) of CTC-MCC-41 cells than hypermethylated CpGs (13,468 CpGs; 17% of the DMCpGs at TFBS). In addition, a small proportion of the CpGs (< 5%) located at enhancer regions showed methylation differences between CTC-MCC-41 and COLO205 cells (Supplementary Fig. 1).

### CTC-MCC-41 cells present a different DNA methylation profile at CpGIs and shore regions of gene promoters than metastatic COLO205 tumor cells

**Figure 4:**
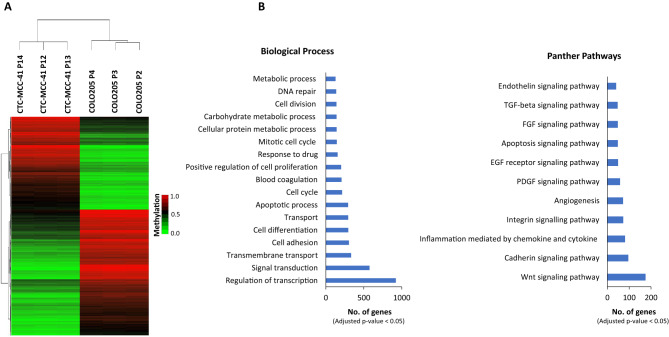
DNA methylation patterns of CpGIs and shore regions of gene promoters in CTC-MCC-41 compared to COLO205 metastatic tumor cells. (**A**) Hierarchical clustering heatmap of the 10,000 most differentially methylated CpGs (FDR adjusted p value < 0.05) in CTC-MCC-41 with respect to COLO205 and located at CpGIs and shore regions of gene promoters. Heatmap shows three different passages (P) of CTC-MCC-41 (P12, P13 and P14) and COLO205 (P2, P3 and P4). (**B**) Gene Ontology (GO) analysis representing some of the most cancer-relevant biological processes and Panther pathways based on the 10,000 most differentially methylated CpGs of CTC-MCC-41 compared to COLO205 and located at CpGIs and shore regions of gene promoters.

When we focused the analysis of CTC-MCC-41 and COLO205 at CpGIs and shores of gene promoters, we were able to identify 26,828 DMCpGs (Fig. 3A), which corresponded with 10,569 genes. Using hierarchical clustering analysis, we observed that the CpGIs and shores of gene promoters in CTC-MCC-41 display a distinctive methylation profile in comparison with the COLO205 metastatic cancer cells (Fig. 4A). Supplementary Table 10 shows the 50 DMCpGs located at these regulatory regions. On the other hand, the GO analysis of the differentially methylated genes identified showed the enrichment of key biological processes, including regulation of transcription, cell adhesion and differentiation, apoptosis, cell cycle and proliferation, metabolic processes and DNA repair. In addition, the Panther pathway analysis revealed an enrichment of important pathways related to cancer development and metastasis, such as Wnt signaling, cadherin pathway, inflammation, angiogenesis and integrin signaling, among others (Fig. 4B and Supplementary Fig. 3).

### CTC-MCC-41 cells have a DNA methylation profile different from both primary and metastatic cancer cells

**Figure 5:**
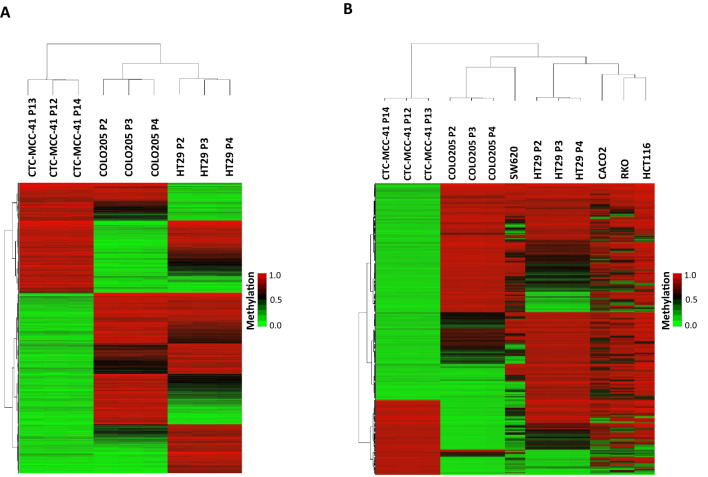
DNA methylation signature of CTC-MCC-41 with respect to colorectal primary and metastatic tumor cells. (**A**) Hierarchical clustering heatmap with the 17,827 differentially methylated CpGs (FDR adjusted p value < 0.05) in CTC-MCC-41 cells compared to HT29 and COLO205 cells, representing primary and metastatic tumor cells, respectively. (**B**) Hierarchical clustering heatmap of the 9,949 differentially methylated CpGs (FDR adjusted p value < 0.05) in CTC-MCC-41 compared to several colorectal primary (HT29, Caco2, HCT116, RKO) and metastatic tumor cells (COLO205 and SW620). Heatmaps show three different passages (P) of CTC-MCC-41 (P12, P13 and P14), HT29 (P2, P3 and P4), and COLO205 (P2, P3 and P4).

After comparing the DNA methylation profile of CTC-MCC-41 with HT29 and COLO205, we wondered whether CTC-MCC-41 could have a methylation profile different from both types of CRC cells (primary and metastatic). Thus, considering the DMCpGs previously identified in CTC-MCC-41 with respect to HT29 and COLO205, a hierarchical clustering analysis revealed an epigenetic signature of 17,827 CpGs that enabled the distinction of CTC-MCC-41 from both HT29 and COLO205 cancer cells (Fig. 5A). Importantly, the tree-based dendrogram yielded two arms: one with CTC-MCC-41 and the other with the primary and metastatic cancer cells.

To confirm the existence of a unique CTC methylation signature in CRC, we extended our analysis to other CRC cell lines representing primary (Caco2, HCT116, RKO) and distant metastatic (SW620) tumors. First, we analyzed the methylation levels of these cancer cell lines with the EPIC array system, and we obtained the DMCpGs (Δβ-value > |0.20|) with respect to CTC-MCC-41. Then, considering the DMCpGs obtained in CTC-MCC-41 with respect to all primary (HT29, Caco2, HCT116, RKO) and metastatic (COLO205 and SW620) cancer cells analysed, we obtained a methylation profile of 9,949 CpGs that enabled the classification of CRC cells according to their cell type: CTC, primary or metastatic cancer cells (Fig. 5B). In particular, the tree-based dendrogram exhibited two clear arms: CTC-MCC-41 was segregated in one branch, and the other cancer cells were segregated in another branch, with two well-differentiated groups containing all the primary cancer cells (HT29, Caco2, HCT116, RKO) and the metastatic cells (COLO205 and SW620).

### The transcriptional program of CTC-MCC-41 cells is epigenetically regulated by DNA methylation

**Figure 6:**
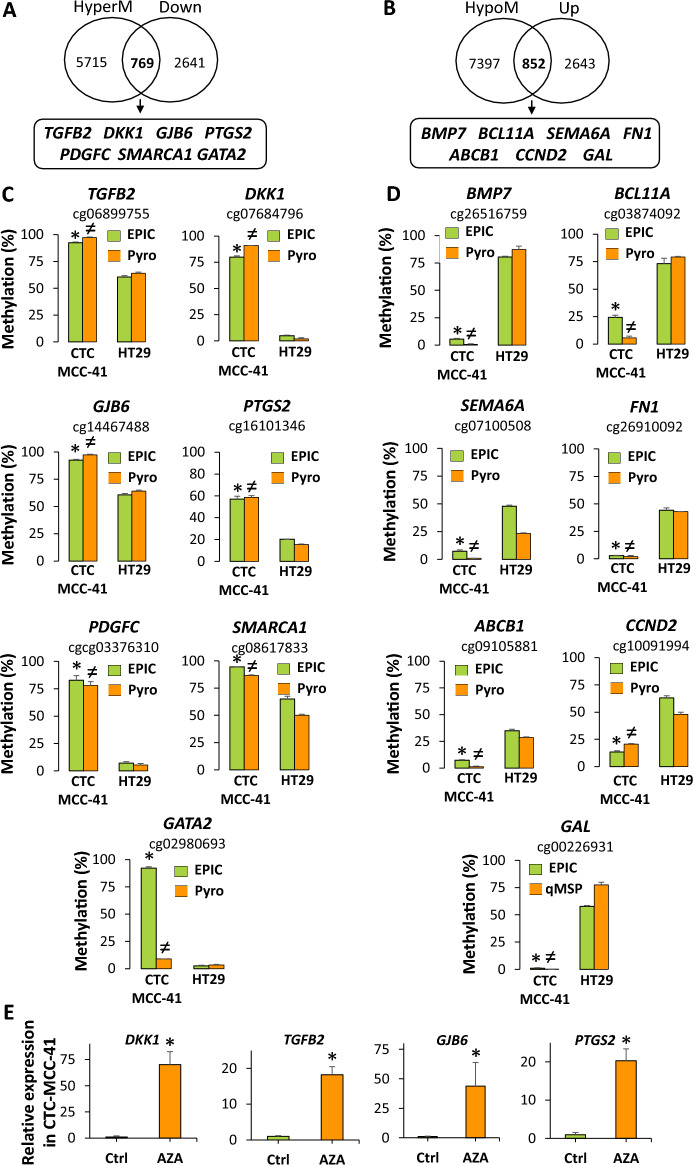
Impact of DNA methylation on the transcriptional program of CTC-MCC-41. (**A**, **B**) Association between the (**A**) hypermethylated/downregulated genes and the (**B**) hypomethylated/upregulated genes found in CTC-MCC-41 with respect to HT29 after the analysis with DNA methylation (EPIC) and expression arrays. HyperM, hypermethylated; HypoM, hypomethylated; Down, downregulated; Up, upregulated. (**C**, **D**) Validation of the methylation status of representative promoter CpGs from selected (**C**) hypermethylated/downregulated genes and (**D**) hypomethylated/upregulated genes. All genes were analyzed by pyrosequencing except *GAL*, which was analyzed by qMSP. EPIC array data are expressed as % of methylation. *p value < 0.05 between CTC-MCC-41 and HT29 after analysis by EPIC array; ^≠^p value < 0.05 between CTC-MCC-41 and HT29 after analysis by pyrosequencing or qMSP. Pyro, pyrosequencing. (**E**) Restored expression of hypermethylated genes (DKK1, TGFB2, *GJB6* and *PTGS2*) in CTC-MCC-41 after treatment with the demethylating agent 5-aza-2′-deoxycytidine (AZA). Gene expression was analyzed by qRT‒PCR. Methylation and expression values were determined from triplicates and are expressed as the mean ± SD. *p value < 0.05 between control (Ctrl) and AZA treatment of CTC-MCC-41 cells.

To determine whether DNA methylation changes at CpGIs and shore regions of gene promoters are able to regulate the gene expression of colorectal CTCs, we took advantage of a previous work published by Alix-Panabières et al*.*^10^, where the transcriptional profile of CTC-MCC-41 was analyzed in comparison with HT29 cancer cells, revealing the differentially expressed genes (up- and downregulated) between both cancer cell types. Thus, we evaluated the association between these differentially expressed genes and the differentially methylated genes in CpGIs and shore regions of gene promoters found in our work when we compared CTC-MCC-41 and HT29 cancer cells (Fig. 6A,B). Venn diagram analysis enabled the identification of 769 genes whose hypermethylation was associated with transcriptional silencing and 852 hypomethylated genes that were associated with transcriptional activation. Importantly, the expression levels of some of the genes identified in our work have been previously validated in CTC-MCC-41 with respect to HT29 by Alix-Panabières et al*.*^10^ (downregulated in CTC-MCC-41: *TGFB2*, *DKK1*, *GJB6*, *PTGS2*, *PDGFC*, *SMARCA1*, and *GATA2*; upregulated in CTC-MCC-41: *BMP7*, *BCL11A*, *SEMA6A*, *FN1*, *ABCB1*, *CCND2*, and *GAL*). Therefore, to confirm the association observed between expression and methylation, we decided to focus our analysis on these previously studied genes and validate the methylation status of their promoter CpGs. Supplementary Tables 11–12 show the methylation levels of the hypermethylated and hypomethylated genes that were obtained in our genome-wide analysis by EPIC array and selected for validation. For the validation assay, we used bisulfite pyrosequencing for all the selected genes except for one (*GAL)*, for which we used qMSP methodology. Importantly, for all the genes analyzed, we were able to confirm the expected presence of hypermethylation or hypomethylation in CTC-MCC-41 with respect to the HT29 cancer cell line. Only in one gene (*GATA2*) was the methylation level observed after bisulfite pyrosequencing in CTC-MCC-41 not as high as that obtained by the EPIC array. Figure 6C and D shows the validation of one representative CpG for each of the selected genes. Other validated CpGs are indicated in Supplementary Fig. 4. After confirming the methylation levels of the selected genes in CTC-MCC-41, we wondered whether the epigenetic program of the hypermethylated genes in colorectal CTCs could be reversed. For this purpose, we treated CTC-MCC-41 cells in vitro with the demethylating agent AZA. Importantly, after AZA treatment, we completely restored the expression of the epigenetically silenced genes *TGFB2*, *DKK1*, *GJB6* and *PTGS2* (Fig. 6E).

In addition to these validated genes, it is noteworthy that among the 852 hypomethylated and downregulated genes identified in CTC-MCC-41, we also found genes involved in pathways relevant to cancer metastasis, such as Wnt signaling (e.g., *FZD5*, *LEF1*, *ACTA2*, *ARRB1*), the cadherin pathway (e.g., *CDH3*, *LYN*, *PCDHA1*, *PCDHA4*, *PCDHA5*, *PCDHA7*, *PCDHA12*), inflammation (e.g., *IKBKB*, *GNG4*, *CASK*) or angiogenesis (e.g., *DLL1*, *LPXN*).

## Discussion

Alteration of epigenetic mechanisms, such as DNA methylation, has relevant implications for CRC development and progression^21^. These epigenetic mechanisms are considered hallmarks of cancer^22^. The analysis of the epigenomic profiles of tumor cells may help to discover new clinical applications and to understand important cancer biological processes, such as metastasis, which is responsible for over 90% of cancer-related deaths^23,24^. CTCs play a causal role in cancer progression, since the dissemination of these cells from primary tumors or metastatic sites may colonize distant tissues and form metastatic tumors^5,6^. Thus, in this study, we compared the DNA methylome of the human colorectal metastasis-competent cell line CTC-MCC-41 with primary (HT29, Caco2, HCT116, RKO) and metastatic (SW620 and COLO205) CRC cell lines, revealing that metastasis-competent CTCs displayed a unique methylation program completely different from those of primary and metastatic tumor cells. To our knowledge, this is the first study that evaluates the DNA methylome of a colon CTC line.

Since the first immortalized cell line (HeLa) was established^25^, human cancer-derived cell lines have been demonstrated to be fundamental models used in laboratories for many years to understand the biology of cancer and pursue clinical applications^21,26,27^. Recent findings based on the characterization of hundreds of cell lines with omics technologies reinforced the concept of cell line usefulness in medical research^28^. In this sense, the epigenomic characterization of CTC lines, such as CTC-MCC-41, represents a great opportunity to provide insights into the biology and clinical applications of this subset of CTCs at the origin of metastatic relapses in cancer patients. CTC-MCC-41 represents a unique model in colon cancer, as it is the only established CTC line obtained from a metastatic patient prior to antitumor therapy^9^.

In the present study, the epigenomic analysis of CTC-MCC-41 revealed that metastasis-competent colorectal CTCs have a different global DNA methylation profile than primary and metastatic cancer cells. It is also noteworthy that we identified that these CTCs are defined by a distinctive methylation signature with respect to primary and metastatic cancer cells. Although the global methylation differences observed were fairly similarly distributed between hypermethylation and hypomethylation, CTCs showed a slight predominance of hypomethylated CpGs. This hypomethylation was mainly distributed in CpG-poor regions, suggesting that the epigenetic regulation of this part of the genome may be important for metastasis-competent CTCs. In cancer, the hypomethylation of these regions has been associated with proto-oncogene expression, chromosomal instability, and malignant transformation of tumors^29,30^. In line with this, other studies have revealed that the cancer cell line CTC-MCC-41^9^ and CTCs obtained from peripheral blood of several tumor types^31,32^ are characterized by the expression of proto-oncogenes and the presence of chromosomal aberrations. On the other hand, with respect to CpGs located at TFBS, we also observed that the number of hypomethylated CpGs associated with TFBS in CTC-MCC-41 was higher than the number of hypermethylated ones. In this sense, a previous work identified that CTCs may undergo methylation changes in CpGs from TFBS^33^. Interestingly, although in a small proportion, some of the methylation differences were located at the CpGs of enhancers, which are epigenetically regulated regions involved in cancer cell plasticity and tumor progression^34^.

A relevant feature of cancer cells is the aberrant methylation of promoter CpGIs and shore regions^11,35^. In our work, we observed that promoter CpGIs and shore regions (CpG-rich promoters) of CTC-MCC-41 are characterized by a different methylation profile than primary and metastatic cancer cells. In this sense, the aberrant promoter methylation of some particular genes (*VIM* and *SFRP2*) has been recently described in CTCs from CRC patients^14^. Importantly, the methylation differences that we found in the promoter CpGI and shore regions of CTCs were highly associated with transcriptional regulation, which is a relevant function of epigenetic mechanisms^11^. Many of these methylation differences were also associated with the Wnt signaling pathway, suggesting that the alteration of this pathway is a relevant characteristic of metastasis-competent CTCs. In many malignancies, including CRC, the alteration of the Wnt pathway is associated with the stemness of cancer cells^36^, which is also a property previously described in CTC-MCC-41 cells^9^. In addition to Wnt signaling, the changes observed in promoter CpGIs and shore regions of CTC-MCC-41 showed association with other relevant biological processes and pathways, including apoptosis, metabolism and DNA repair, among others. Importantly, a previous work in CTC-MCC-41 showed that the transcriptional program of this cell line is associated with changes in the expression of genes involved in these pathways^10^.

In cancer, aberrant DNA methylation of CpGI and shore promoters is usually linked to changes in gene expression, with a negative correlation between methylation and gene expression^21,37^. In line with this, in the present work, multiple genes of CTC-MCC-41 showed a negative association between their DNA methylation and transcriptional levels. Importantly, in several selected genes, we confirmed promoter hypermethylation (*TGFB2*, *DKK1*, *GJB6*, *PTGS2*, *PDGFC*, *SMARCA1*, *GATA2*) or hypomethylation (*BMP7*, *BCL11A*, *SEMA6A*, *FN1*, *ABCB1*, *CCND2*, *GAL*). One important property of DNA methylation is that the silencing of this mechanism can be reversed with epigenetic drugs (epidrugs), such as DNA demethylating agents. In this sense, the 5-aza-2′-deoxycytidine drug was able to reverse the epigenetic silencing of some of the hypermethylated genes in CTC-MCC-41, including *DKK1*, *TGFB2*, *GJB6* and *PTGS2*, confirming the epigenetic regulation of these genes by DNA methylation. Altogether, these results indicate that DNA methylation is a regulator of the transcriptional program in metastasis-competent CTCs.

In addition, to provide relevant biological information, the aberrantly methylated genes identified in this work also represent potential biomarkers and therapeutic targets for metastasis-competent CTCs. For example, we have identified the hypermethylation of *DKK1*, which is a tumor suppressor gene that works as a potent inhibitor of the canonical Wnt signaling pathway to avoid epithelial-mesenchymal transition, cell proliferation and metastasis in cancer^38,39^. *DKK1* has been found to be hypermethylated in several types of tumors, and its tumor suppressor activity can be reversed using demethylation agents^40,41^, suggesting that hypermethylation of this gene may be a new therapeutic target in metastatic-competent CTCs. In line with this, the use of DNA demethylating drugs, such as guadecitabine (SGI-110), is under study in metastatic CRC patients^42^. On the other hand, the hypermethylation of *DKK1* has also been identified as a prognostic factor in cancer patients^43,44^, indicating that the hypermethylation of this gene in CTCs could have clinical implications as a prognostic biomarker.

Importantly, we have identified other potential therapeutic targets in metastasis-competent CTCs that are involved in the activation of the Wnt signaling pathway. This is the case for the genes *FZD5*, *LEF1*, *ACTA2*, and *ARRB1,* which showed transcriptional activation associated with hypomethylation in the CTC-MCC-41 cell line. Importantly, therapeutic targeting of the Wnt pathway is a promising strategy under study to impair cancer cells in several tumors, including CRC^45,46^. This is the case, for example, for vantictumab (OMP-18R5), which is a fully human monoclonal antibody that inhibits the Wnt pathway through binding of several FZD receptors, including FZD5^47^, which has been evaluated in clinical trials with solid tumors (NCT01973309, NCT01957007, NCT02005315). In line with this, the hypomethylation of *FZD5* could be a potential biomarker to select patients who would benefit from anti-FZD5 therapies. Similarly, the inhibition of LEF1, which is a transcription factor with a central role in the Wnt pathway, has shown antitumoral properties in several preclinical studies^48^. In line with this, we have identified in CTC-MCC-41 cells other promoter hypomethylated genes involved in the cadherin pathway (*DH3*, *LYN*, *PCDHA1*, *PCDHA4*, *PCDHA5*, *PCDHA7*, *PCDHA12*), inflammation (*IKBKB*, *GNG4*, *CASK*) or angiogenesis (*DLL1*, *LPXN*), suggesting that these genes could also be therapeutic targets for metastasis-competent CTCs in CRC patients.

It has been described that the survival duration of metastasis-competent CTCs in the vascular system represents one of the most critical parameters that controls the formation of clinical metastases^4^. In this sense, for example, the activation of Wnt signaling pathway in colorectal tumor cells stimulates stemness properties, survival and proliferation^49^; suggesting that the activation of this pathway by deregulated epigenetic mechanisms in metastasis-competent CTCs could promote the survival of these cells in circulation. Therefore, the use of therapies that target Wnt signaling pathway could be a relevant approach to reduce the survival of this type of CTCs in the vascular system, which could significantly reduce the risk of metastasis^4^.

The epigenetically deregulated genes identified in the metastasis-competent CTCs analyzed in this work open the possibility to target and eradicate this subtype of CTCs with new drugs or with a combination of existing ones. In future studies, it would be interesting to evaluate the epigenetically deregulated genes found in this work in peripheral CTCs from CRC patients.

## Conclusions

The characterization of the epigenomic profile of CTC-MCC-41 revealed that metastasis-competent CTCs in CRC display a globally different DNA methylation program than primary and metastatic tumor cells. These CTCs also have a unique DNA methylation profile of their CpG-rich promoters that is associated with relevant cancer pathways and has the ability to regulate the transcriptional programs of these cells. The aberrantly DNA methylated genes identified in this work represent potential biomarkers and therapeutic targets of metastasis-competent CTCs that may contribute to the development of new specific therapies directed against this type of CTCs in CRC.

### Supplementary Information

Supplementary Information.

## Data Availability

The DNA methylation data obtained with EPIC array in this study are publicly available at the Gene Expression Omnibus (GEO) repository with the Accession Number GSE220096.

## Abbreviations

AZA: 5-Aza-2′-deoxycytidine
DMCpGs: Differentially methylated CpGs
CpG: Cytosine-phosphate-guanine
CpGI: CpG island
CRC: Colorectal cancer
CTC: Circulating tumor cells
TFBS: Transcription factor-binding sites
