# Publisher Correction: Epigenomic analysis reveals a unique DNA methylation program of metastasis-competent circulating tumor cells in colorectal cancer

**DOI:** 10.1038/s41598-023-43747-x

**Published:** 2023-10-09

**Authors:** Aida Bao-Caamano, Nicolás Costa-Fraga, Laure Cayrefourcq, María Amalia Jácome, Aitor Rodriguez-Casanova, Laura Muinelo-Romay, Rafael López-López, Catherine Alix-Panabières, Angel Díaz-Lagares

**Affiliations:** 1 Epigenomics Unit, Cancer Epigenomics, Translational Medical Oncology Group (ONCOMET), Health Research Institute of Santiago de Compostela (IDIS), University Clinical Hospital of Santiago (CHUS/SERGAS), 15706 Santiago de Compostela, Spain; 2 Universidade de Santiago de Compostela (USC), 15782 Santiago de Compostela, Spain; 3 Galician Precision Oncology Research Group (ONCOGAL), Medicine and Dentistry School, Universidade de Santiago de Compostela (USC), Santiago de Compostela, Spain; 4 Laboratory of Rare Human Circulating Cells, University Medical Center of Montpellier, IURC, 641, Avenue du Doyen Gaston Giraud, 34093 Montpellier Cedex 5, France; 5 CREEC, MIVEGEC, University of Montpellier, CNRS, IRD, Montpellier, France; 6 Department of Mathematics, MODES Group, CITIC, Faculty of Science, Universidade da Coruña, A Coruña, Spain; 7 Roche-Chus Joint Unit, Translational Medical Oncology Group (ONCOMET), Health Research Institute of Santiago (IDIS), 15706 Santiago de Compostela, Spain; 8 Liquid Biopsy Analysis Unit, Translational Medical Oncology Group (ONCOMET), Health Research Institute of Santiago de Compostela (IDIS), 15706 Santiago de Compostela, Spain; 9 Centro de Investigación Biomédica en Red Cáncer (CIBERONC), ISCIII, 28029 Madrid, Spain; 10 Translational Medical Oncology Group (ONCOMET), Health Research Institute of Santiago de Compostela (IDIS), University Clinical Hospital of Santiago (CHUS/SERGAS), 15706 Santiago de Compostela, Spain; 11 European Liquid Biopsy Society (ELBS), Hamburg, Germany; 12 Department of Clinical Analysis, University Hospital Complex of Santiago de Compostela (CHUS), 15706 Santiago de Compostela, Spain

Correction to: *Scientific Reports* 10.1038/s41598-023-42037-w, published online 16 September 2023

The original HTML version of this Article contained errors in Figures 1 to 5, where the dendrograms were not displayed correctly.

The original Figures 1, 2, 3, 4 and 5 and their accompanying legends appear below.

**Figure 1:**
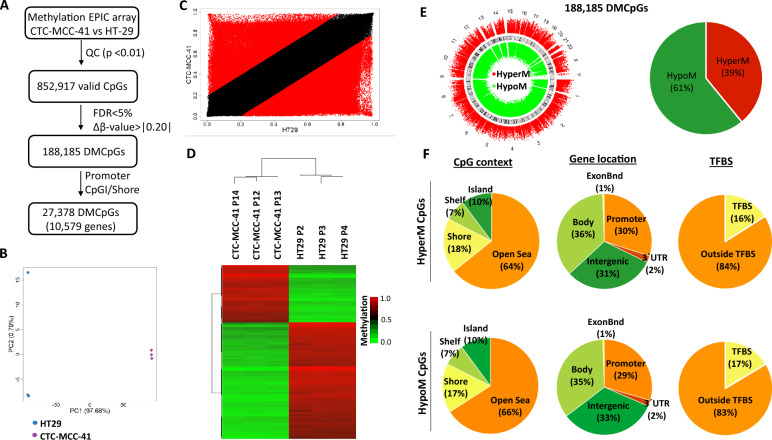
Genome-wide DNA methylation analysis of CTC-MCC-41 with respect to HT29 primary tumor cells. (**A**) Schematic flowchart used to identify significant differentially methylated CpGs in CTC-MCC-41 compared to HT29. (**B**) Principal component analysis (PCA) of DNA methylation data obtained in CTC-MCC-41 and HT29 cells. (**C**) Scatter plot representing the mean normalized levels of DNA methylation (β-values) in CTC-MCC-41 and HT29 cells. Dots in red show significantly differentially methylated CpGs. (**D**) Hierarchical clustering heatmap of the 10,000 most differentially methylated CpGs (FDR adjusted p value < 0.05) between CTC-MCC-41 and HT29. Heatmap shows three different passages (P) of CTC-MCC-41 (P12, P13 and P14) and HT29 (P2, P3 and P4). (**E**, **F**) Description of the 188,185 differentially methylated CpGs (DMCpGs) found in CTC-MCC-41 with respect to HT29 according to (**E**) chromosome location and methylation status and (**F**) CpG context, gene location and transcription factor-binding sites (TFBS). *QC* quality control, *FDR* false discovery rate, *CpGI* CpG island, *HypoM* hypomethylated, *HyperM* hypermethylated.

**Figure 2:**
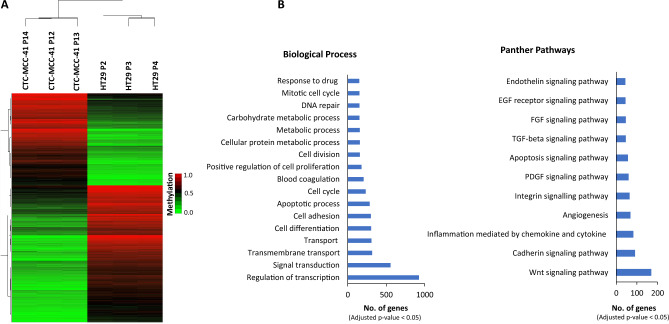
DNA methylation profiles of CpGIs and shore regions of gene promoters in CTC-MCC-41 compared to HT29 primary tumor cells. (**A**) Hierarchical clustering heatmap of the 10,000 most differentially methylated CpGs (FDR adjusted p value < 0.05) in CTC-MCC-41 with respect to HT29 and located at CpGIs and shore regions of gene promoters. Heatmap shows three different passages (P) of CTC-MCC-41 (P12, P13 and P14) and HT29 (P2, P3 and P4). (**B**) Gene Ontology (GO) analysis representing some of the most cancer-relevant biological processes and Panther pathways based on the 10,000 most differentially methylated CpGs of CTC-MCC-41 compared to HT29 and located at CpGIs and shore regions of gene promoters.

**Figure 3:**
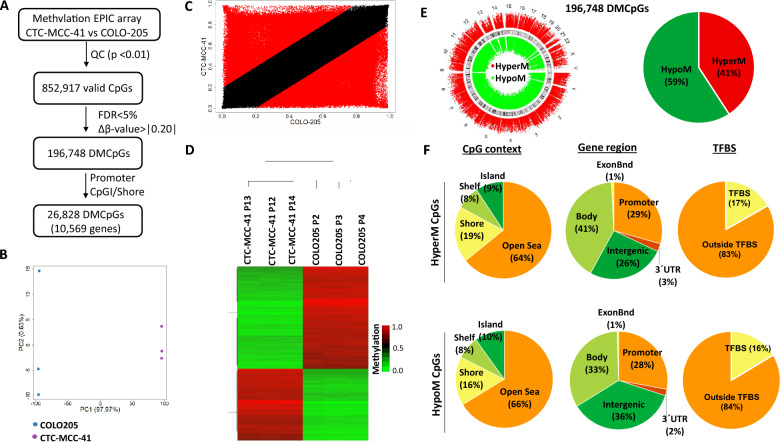
Genome-wide DNA methylation analysis of CTC-MCC-41 with respect to COLO205 metastatic tumor cells. (**A**) Schematic flowchart used to identify significantly differentially methylated CpGs in CTC-MCC-41 compared to COLO205. (**B**) Principal component analysis (PCA) of DNA methylation data obtained for CTC-MCC-41 and COLO205 cells. (**C**) Scatter plot representing the mean normalized levels of DNA methylation (β-values) in CTC-MCC-41 and COLO205 cells. Dots in red show significantly differentially methylated CpGs. (**D**) Hierarchical clustering heatmap of the 10,000 most differentially methylated CpGs (FDR adjusted p value < 0.05) between CTC-MCC-41 and COLO205. Heatmap shows three different passages (P) of CTC-MCC-41 (P12, P13 and P14) and COLO205 (P2, P3 and P4). (**E**, **F**) Description of the 188,185 differentially methylated CpGs (DMCpGs) found in CTC-MCC-41 with respect to COLO205 according to (**E**) chromosome location and methylation status and (**F**) CpG context, gene location and transcription factor-binding sites (TFBS). *QC* quality control, *FDR* false discovery rate, *CpGI* CpG island, *HypoM* hypomethylated, *HyperM* hypermethylated.

**Figure 4:**
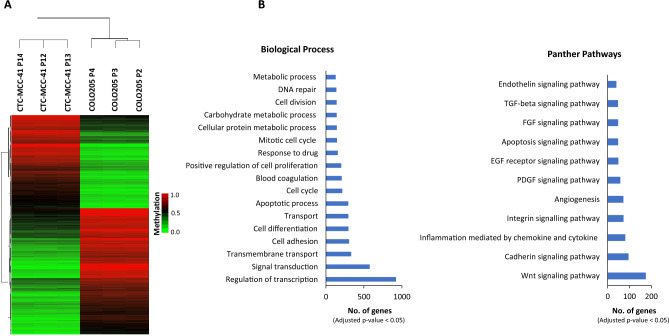
DNA methylation patterns of CpGIs and shore regions of gene promoters in CTC-MCC-41 compared to COLO205 metastatic tumor cells. (**A**) Hierarchical clustering heatmap of the 10,000 most differentially methylated CpGs (FDR adjusted p value < 0.05) in CTC-MCC-41 with respect to COLO205 and located at CpGIs and shore regions of gene promoters. Heatmap shows three different passages (P) of CTC-MCC-41 (P12, P13 and P14) and COLO205 (P2, P3 and P4). (**B**) Gene Ontology (GO) analysis representing some of the most cancer-relevant biological processes and Panther pathways based on the 10,000 most differentially methylated CpGs of CTC-MCC-41 compared to COLO205 and located at CpGIs and shore regions of gene promoters.

**Figure 5:**
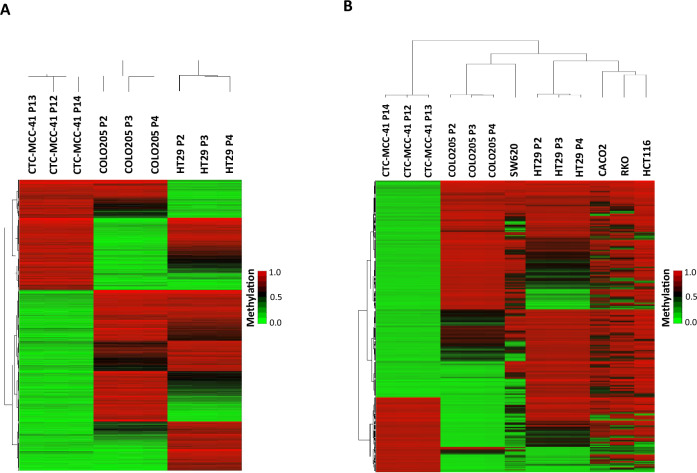
DNA methylation signature of CTC-MCC-41 with respect to colorectal primary and metastatic tumor cells. (**A**) Hierarchical clustering heatmap with the 17,827 differentially methylated CpGs (FDR adjusted p value < 0.05) in CTC-MCC-41 cells compared to HT29 and COLO205 cells, representing primary and metastatic tumor cells, respectively. (**B**) Hierarchical clustering heatmap of the 9,949 differentially methylated CpGs (FDR adjusted p value < 0.05) in CTC-MCC-41 compared to several colorectal primary (HT29, Caco2, HCT116, RKO) and metastatic tumor cells (COLO205 and SW620). Heatmaps show three different passages (P) of CTC-MCC-41 (P12, P13 and P14), HT29 (P2, P3 and P4), and COLO205 (P2, P3 and P4).

These errors have now been corrected in the HTML version of the Article; the PDF version was correct from the time of publication.

