# Supplementary material for: Epigenomic analysis reveals a unique DNA methylation program of metastasis-competent circulating tumor cells in colorectal cancer
Source: Sci Rep. 2023 Sep 16;13:15401. doi: 10.1038/s41598-023-42037-w (PMC10505142; doi:10.1038/s41598-023-42037-w)
Supplement: Supplementary file 1 — Supplementary Information. [file 41598_2023_42037_MOESM1_ESM.docx]

**Supplementary Tables/Figures**

**Supplementary Table 1.** General characteristics of the human colorectal cancer cell lines used in the study for DNA methylation analysis.

| **Cell line** | **ATCC ID** | **Disease** | **Origin** | **Age** | **Gender** |
| --- | --- | --- | --- | --- | --- |
| **HT29** | HTB-38 | Colorectal adenocarcinoma | Primary tumor (colon) | 44-year-old | Female |
| **Caco2** | HTB-37 | Colorectal adenocarcinoma | Primary tumor (colon) | 72-year-old | Male |
| **HCT116** | CCL-247 | Colorectal carcinoma | Primary tumor (colon) | 48-year-old | Male |
| **RKO** | CRL-2577 | Colorectal carcinoma | Primary tumor (colon) | - | - |
| **COLO205** | CCL-222 | Colorectal cdenocarcinoma (Dukes´ type D) | Metastasis (ascitic fluid) | 70-year -old | Male |
| **SW620** | CCL-227 | Colorectal adenocarcinoma (Dukes' type C) | Metastasis (lymph node) | 51-year -old | Male |
| **CTC-MCC-41** | - | Colorectal adenocarcinoma | Circulating tumour cell (peripheral Blood) | 57-year -old | Male |

**Supplementary Table 2.** Primer sequences for DNA methylation analysis by pyrosequencing.

| **Gene** | **Primer** | **Sequence (5´- 3´)** |
| --- | --- | --- |
| ***ABCB1*** | Forward | [Btn] GGTTGATTGGTTGGGTAGGAATA |
|  | Reverse | ACACTTAAAAACTATCCCATAATAACTC |
|  | Sequencing | ATCCCATAATAACTCCC |
| ***BCL11A*** | Forward | [Btn] TGGATGTTAAAAGGTATTGATGAAGATA |
|  | Reverse | TCTCCCCCTCRCTTTTACTTCTAATCCT |
|  | Sequencing | ACTTAAATACTAAAATTTACCTTA |
| ***BMP7*** | Forward | TGGTYGATTTTAGTTTGGATAA |
|  | Reverse | [Btn] ATAAATACCRAATTATACTTACC |
|  | Sequencing | ATTTTTTTTATTTTGGGTTTGTT |
| ***CCND2*** | Forward | GGGAGGAAGGAGGTGAAGAAA |
|  | Reverse | [Btn] CACCACCCCTTCCTTTCC |
|  | Sequencing | TTTGTAAAGATAGTTTTGATTTAAG |
| ***DKK1*** | Forward | TGGGGATTTAGGTTTGTAAAGTGA |
|  | Reverse | [Btn] AATTCAAAATAACRCTCACTCCCAACAA |
|  | Sequencing | TTTGAGTTTTTTTGAGATGAT |
| ***FN1*** | Forward | GGAAGAGGGGATTTGTAGTTATAATT |
|  | Reverse | [Btn] ACAAATTACCACCAAATTTACTTCC |
|  | Sequencing | AGGTATTAGAAGGGATTTT |
| ***GATA2*** | Forward | [Btn] GGGTAGGGGATTTAGTTAGG |
|  | Reverse | AACAAATAATTCYCCTTTTTCTCCCAC |
|  | Sequencing | ATTACCCTCCCCCTC |
| ***GJB6*** | Forward | TTAAGGGTYGGAAAGAGG |
|  | Reverse | [Btn] AACCYAAACTATCCCTCC |
|  | Sequencing | TGTATTTTTTAGGGGTTTTTTAT |
| ***PDGFC*** | Forward | GTTTTGGATGGGATTATGTGGA |
|  | Reverse | [Btn] CCTAAATCCCCTATCTCTAACC |
|  | Sequencing | ATGGGATTATGTGGAAA |
| ***PTGS2*** | Forward | AGGGAGAGAAATGTTTTAAGGT |
|  | Reverse | [Btn] TCACCAATATAAAATTTCCTACCTTCTAA |
|  | Sequencing | GGAGAGAAATGTTTTAAGGTATA |
| ***SEMA6A*** | Forward | AGATAAGTATATATGTAGGAATGATAAAGG |
|  | Reverse | [Btn] CCCTCAAACTAAAATCCAACTACC |
|  | Sequencing | ATAATGGATTATTAAATGGGATATA |
| ***SMARCA1*** | Forward | AGGGGAGTTTAGGTTTATTGTTGG |
|  | Reverse | [Btn] TCCCCTTCCCCACCCAAAA |
|  | Sequencing | GTGGGAAAGGAGATT |
| ***TGFB2*** | Forward | [Btn] ATTAGGGATTTGTTTTAGGAGAAG |
|  | Reverse | ACATATCTATTTTATAAACCTCCT |
|  | Sequencing | CATATCTATTTTATAAACCTCCTT |

Btn, biotin

| **Gene** | **Methylation status** | **Primer** | **Sequence (5´- 3´)** |
| --- | --- | --- | --- |
| ***GAL*** | Methylation | Forward | GTGAGCGTTTTAGGTCGTTA |
|  |  | Reverse | ACTACGACTACGCCCGAAT |
|  | Unmethylation | Forward | GTGAGTGTTTTAGGTTGTTAGAGTT |
|  |  | Reverse | CTTAAACTACAACTACACCCAAAT |

**Supplementary Table 3.** Primer sequences used for the qMSP analysis.

**Supplementary Table 4**. Primer sequences for the qRT-PCR analysis**.**

| **Gene** | **Primer** | **Sequence (5´-3´)** |
| --- | --- | --- |
| ***DKK1*** | Forward | GGTATTCCAGAAGAACCACCTTG |
|  | Reverse | CTTGGACCAGAAGTGTCTAGCAC |
| ***GJB6*** | Forward | GAAACCACTCGCAAGTTCAGGC |
|  | Reverse | AGGCTGCTTCAAAGATGATTCGG |
| ***PDGFC*** | Forward | TGAACCAGGGTTCTGCATCCAC |
|  | Reverse | TAAGCAGGTCCAGTGGCAAAGC |
| ***PTGS2*** | Forward | CGGTGAAACTCTGGCTAGACAG |
|  | Reverse | GCAAACCGTAGATGCTCAGGGA |
| ***SMARCA1*** | Forward | AACCTGGCAAGTGCTGATGTGG |
|  | Reverse | CAGTGTTGTCAGTGATGAGACGG |
| ***TGFB2*** | Forward | AAGAAGCGTGCTTTGGATGCGG |
|  | Reverse | ATGCTCCAGCACAGAAGTTGGC |
| ***B2M*** | Forward | GTCTTTCAGCAAGGACTGGTCT |
|  | Reverse | TTACATGTCTCGATCCCACTTAAC |

**Supplementary Table 5.** Top 50 differentially methylated CpGs identified in CTC-MCC-41 in comparison to HT29 cells.

| **TargetID^1^** | **Chr^2^** | **Position** | **Gene name** | **Gene region** | **CpG context** | **Δβ^3^** |
| --- | --- | --- | --- | --- | --- | --- |
| cg19127343 | 19 | 57106620 | *ZNF71* | TSS200 | Island | -0.979 |
| cg11111131 | X | 105855108 | *CXorf57* | TSS200 | Island | -0.977 |
| cg19398192 | X | 105855052 | *CXorf57* | TSS200 | Island | -0.977 |
| cg01145140 | 6 | 166077613 |  |  | Island | -0.975 |
| cg03304380 | 8 | 13134144 | *DLC1* | Body | Island | -0.975 |
| cg23216956 | 19 | 57106627 | *ZNF71* | TSS200 | Island | -0.973 |
| cg08935899 | 11 | 6651712 | *DCHS1* | Body | Island | -0.973 |
| cg09744766 | 16 | 53407421 |  |  | Island | 0.973 |
| cg08812555 | 10 | 54074788 | *DKK1* | Body | Island | 0.972 |
| cg25492241 | 3 | 159482591 | *SCHIP1* | Body | Island | 0.971 |
| cg25230111 | 19 | 56159817 | *CCDC106* | 5'UTR | Island | 0.971 |
| cg13633361 | 12 | 127631167 |  |  | Island | -0.971 |
| cg24447438 | 18 | 21718979 | *CABYR;CABYR;CABYR;CABYR;CABYR;CABYR;CABYR;CABYR;CABYR;CABYR;CABYR* | 1stExon;1stExon;1stExon;5'UTR;5'UTR;5'UTR;TSS1500;1stExon;5'UTR;5'UTR;1stExon | Island | -0.970 |
| cg11739758 | 17 | 46670663 | *LOC404266;LOC404266;LOC404266;HOXB5;LOC404266* | Body;Body;Body;1stExon;Body | Island | 0.970 |
| cg11757144 | 7 | 91510093 | *MTERF* | TSS200 | Island | 0.969 |
| cg07030010 | 6 | 74405929 | *CD109;CD109;CD109;CD109;CD109;CD109;LOC101928489* | 5'UTR;5'UTR;5'UTR;1stExon;1stExon;1stExon;TSS200 | Island | -0.969 |
| cg21159778 | 9 | 117266918 | *DFNB31;DFNB31* | TSS1500;1stExon | Island | -0.969 |
| cg27586390 | X | 105855176 | *CXorf57;CXorf57* | 1stExon;5'UTR | Island | -0.969 |
| cg18496937 | 22 | 19702076 | *SEPT5;SEPT5* | 1stExon;5'UTR | Shore | -0.967 |
| cg04210573 | X | 71351784 | *RGAG4;NHSL2* | TSS200;Body | Island | -0.967 |
| cg02728595 | 3 | 196255632 |  |  | Island | -0.966 |
| cg07735777 | 7 | 140715095 | *MRPS33;MRPS33* | TSS1500;TSS1500 | Shore | 0.966 |

**Supplementary Table 5.** *Continued*

| **TargetID^1^** | **Chr^2^** | **Position** | **Gene name** | **Gene region** | **CpG context** | **Δβ^3^** |
| --- | --- | --- | --- | --- | --- | --- |
| cg01571001 | 3 | 128215433 |  |  | Island | -0.966 |
| cg00740547 | 21 | 46439236 |  |  | Island | -0.965 |
| cg00018261 | X | 117957689 | *ZCCHC12* | TSS200 | Island | -0.965 |
| cg25249518 | 6 | 119399928 | *FAM184A;FAM184A* | 5'UTR;TSS200 | Island | -0.965 |
| cg20081969 | 15 | 80697055 | *ARNT2* | Body | Island | -0.965 |
| cg11127711 | 12 | 59990120 |  |  | Island | 0.965 |
| cg26777760 | X | 132092564 | *HS6ST2;HS6ST2* | Body;Body | Island | -0.964 |
| cg19452316 | 1 | 206680966 | *RASSF5;RASSF5* | 1stExon;1stExon | Island | -0.964 |
| cg03899721 | 8 | 8748792 | *MFHAS1* | 1stExon | Island | 0.964 |
| cg10594237 | 3 | 183165594 |  |  | Island | -0.963 |
| cg11218091 | 10 | 124638976 | *LOC399815;FAM24B* | TSS200;5'UTR | Island | -0.962 |
| cg02737782 | 1 | 8014393 |  |  | Island | 0.962 |
| cg05168842 | 11 | 61584319 | *FADS1* | 1stExon | Island | -0.962 |
| cg26156167 | 1 | 161582651 |  |  | Shore | 0.962 |
| cg21070172 | 22 | 46932833 | *CELSR1* | 1stExon | Island | 0.961 |
| cg01774894 | 17 | 46674395 | *LOC404266;LOC404266;LOC404266;LOC404266;HOXB6;LOC404266* | Body;Body;Body;Body;Body;Body | Shore | 0.961 |
| cg06791102 | X | 153191600 | *ARHGAP4;ARHGAP4* | 1stExon;1stExon | Island | -0.961 |
| cg03679269 | X | 71351803 | *NHSL2;RGAG4* | Body;TSS200 | Island | -0.961 |
| cg06377278 | 1 | 25256369 | *RUNX3;RUNX3;RUNX3* | 1stExon;Body;5'UTR | Island | -0.960 |
| cg18647259 | 17 | 36610306 |  |  | Island | 0.960 |
| cg14057181 | X | 105855056 | *CXorf57* | TSS200 | Island | -0.960 |
| cg01986767 | 16 | 50425351 |  |  |  | -0.959 |
| cg11088422 | 17 | 42392697 | *RUNDC3A;RUNDC3A;RUNDC3A* | Body;Body;Body | Island | 0.959 |
| cg01632288 | 19 | 12880789 | *HOOK2;HOOK2* | Body;Body | Island | -0.959 |

**Supplementary Table 5.** *Continued*

| **TargetID^1^** | **Chr^2^** | **Position** | **Gene name** | **Gene region** | **CpG context** | **Δβ^3^** |
| --- | --- | --- | --- | --- | --- | --- |
| cg09247193 | 17 | 47075095 | *IGF2BP1;IGF2BP1;IGF2BP1;IGF2BP1* | 5'UTR;5'UTR;1stExon;1stExon | Island | -0.959 |
| cg01345354 | 6 | 2892152 | *SERPINB9* | Body | Island | -0.958 |
| cg17801765 | 1 | 958274 | *AGRN* | Body | Shore | 0.958 |
| cg12253469 | 22 | 37420454 | *MPST;MPST;MPST;MPST* | Body;Body;Body;Body | Island | -0.957 |

^1^Identification of the CpG according to EPIC array; ^2^Chromosome; ^3^Δβ-values (β-value CTC-MCC-41 - β-value HT29). All CpGs in the table are significant (FDR adjusted p value < 0.05) and are arranged according to their absolute Δβ-value.

**Supplementary Table 6.** Top 50 differentially methylated CpGs from CpG islands or shore regions of gene promoters identified in CTC-MCC-41 in comparison to HT29 cells.

| **TargetID^1^** | **Chr^2^** | **Position** | **Gene name** | **Gene region** | **CpG context** | **Δβ^3^** |
| --- | --- | --- | --- | --- | --- | --- |
| cg19127343 | 19 | 57106620 | *ZNF71* | TSS200 | Island | -0.979 |
| cg11111131 | X | 105855108 | *CXorf57* | TSS200 | Island | -0.977 |
| cg19398192 | X | 105855052 | *CXorf57* | TSS200 | Island | -0.977 |
| cg23216956 | 19 | 57106627 | *ZNF71* | TSS200 | Island | -0.973 |
| cg25230111 | 19 | 56159817 | *CCDC106* | 5'UTR | Island | 0.971 |
| cg24447438 | 18 | 21718979 | *CABYR;CABYR;CABYR;CABYR;CABYR;CABYR;CABYR;CABYR;CABYR;CABYR;CABYR* | 1stExon;1stExon;1stExon;5'UTR;5'UTR;5'UTR;TSS1500;1stExon;5'UTR;5'UTR;1stExon | Island | -0.970 |
| cg11739758 | 17 | 46670663 | *LOC404266;LOC404266;LOC404266;HOXB5;LOC404266* | Body;Body;Body;1stExon;Body | Island | 0.970 |
| cg11757144 | 7 | 91510093 | *MTERF* | TSS200 | Island | 0.969 |
| cg07030010 | 6 | 74405929 | *CD109;CD109;CD109;CD109;CD109;CD109;LOC101928489* | 5'UTR;5'UTR;5'UTR;1stExon;1stExon;1stExon;TSS200 | Island | -0.969 |
| cg21159778 | 9 | 117266918 | *DFNB31;DFNB31* | TSS1500;1stExon | Island | -0.969 |
| cg27586390 | X | 105855176 | *CXorf57;CXorf57* | 1stExon;5'UTR | Island | -0.969 |
| cg18496937 | 22 | 19702076 | *SEPT5;SEPT5* | 1stExon;5'UTR | Shore | -0.967 |
| cg04210573 | X | 71351784 | *RGAG4;NHSL2* | TSS200;Body | Island | -0.967 |
| cg07735777 | 7 | 140715095 | *MRPS33;MRPS33* | TSS1500;TSS1500 | Shore | 0.966 |
| cg00018261 | X | 117957689 | *ZCCHC12* | TSS200 | Island | -0.965 |
| cg25249518 | 6 | 119399928 | *FAM184A;FAM184A* | 5'UTR;TSS200 | Island | -0.965 |
| cg19452316 | 1 | 206680966 | *RASSF5;RASSF5* | 1stExon;1stExon | Island | -0.964 |
| cg03899721 | 8 | 8748792 | *MFHAS1* | 1stExon | Island | 0.964 |

**Supplementary Table 6.** *Continued*

| cg11218091 | 10 | 124638976 | *LOC399815;FAM24B* | TSS200;5'UTR | Island | -0.962 |
| --- | --- | --- | --- | --- | --- | --- |
| cg05168842 | 11 | 61584319 | *FADS1* | 1stExon | Island | -0.962 |
| cg21070172 | 22 | 46932833 | *CELSR1* | 1stExon | Island | 0.961 |
| cg06791102 | X | 153191600 | *ARHGAP4;ARHGAP4* | 1stExon;1stExon | Island | -0.961 |
| cg03679269 | X | 71351803 | *NHSL2;RGAG4* | Body;TSS200 | Island | -0.961 |
| cg06377278 | 1 | 25256369 | *RUNX3;RUNX3;RUNX3* | 1stExon;Body;5'UTR | Island | -0.960 |
| cg14057181 | X | 105855056 | *CXorf57* | TSS200 | Island | -0.960 |
| cg09247193 | 17 | 47075095 | *IGF2BP1;IGF2BP1;IGF2BP1;IGF2BP1* | 5'UTR;5'UTR;1stExon;1stExon | Island | -0.959 |
| cg21926402 | 3 | 148804275 | *HLTF;HLTF;HLTF;HLTF* | 5'UTR;1stExon;1stExon;5'UTR | Island | -0.957 |
| cg14465900 | 9 | 132805755 | *FNBP1* | TSS1500 | Island | 0.957 |
| cg21174533 | 14 | 102026555 | *DIO3* | TSS1500 | Island | 0.957 |
| cg23337116 | 4 | 53728510 | *RASL11B;RASL11B* | 1stExon;5'UTR | Island | -0.957 |
| cg24843646 | 6 | 74405650 | *CD109;CD109;CD109;CD109;CD109;CD109* | 5'UTR;1stExon;5'UTR;1stExon;5'UTR;1stExon | Island | -0.955 |
| cg20340302 | 20 | 55841149 | *BMP7* | 1stExon | Island | -0.955 |
| cg06266993 | 17 | 46671131 | *LOC404266;LOC404266;LOC404266;LOC404266;HOXB5* | Body;Body;Body;Body;TSS200 | Island | 0.955 |
| cg01316876 | 6 | 119399931 | *FAM184A;FAM184A;FAM184A* | TSS200;5'UTR;5'UTR | Island | -0.954 |
| cg18328206 | 1 | 206681378 | *RASSF5;RASSF5* | 1stExon;1stExon | Island | -0.953 |
| cg06796204 | X | 71351792 | *NHSL2;RGAG4* | Body;TSS200 | Island | -0.953 |
| cg24928995 | 18 | 21718893 | *CABYR;CABYR;CABYR;CABYR;CABYR;CABYR* | TSS200;TSS200;TSS200;TSS200;TSS1500;TSS200 | Island | -0.953 |
| cg07136888 | 15 | 90358131 | *ANPEP* | TSS200 | Island | -0.953 |

**Supplementary Table 6.** *Continued*

| cg01032200 | 1 | 155290641 | *RUSC1-AS1;RUSC1;RUSC1;RUSC1;RUSC1* | Body;1stExon;1stExon;5'UTR;5'UTR | Island | 0.952 |
| --- | --- | --- | --- | --- | --- | --- |
| cg27173322 | 11 | 61584112 | *MIR1908;FADS1* | TSS1500;1stExon | Island | -0.951 |
| cg16977570 | 13 | 24152974 | *TNFRSF19;TNFRSF19* | TSS1500;5'UTR | Island | -0.951 |
| cg22806837 | 19 | 58011345 | *ZNF773;ZNF773* | 5'UTR;1stExon | Island | 0.951 |
| cg09133006 | 1 | 206680888 | *RASSF5;RASSF5;RASSF5;RASSF5* | 1stExon;5'UTR;5'UTR;1stExon | Island | -0.951 |
| cg07910813 | 18 | 65183745 | *DSEL;DSEL* | 1stExon;5'UTR | Island | 0.950 |
| cg07813249 | 4 | 46392253 | *GABRA2;GABRA2* | TSS200;TSS1500 | Island | -0.950 |
| cg05948940 | 16 | 68481342 | *SMPD3* | 5'UTR | Island | 0.949 |
| cg01388796 | 19 | 3801548 | *MATK;MATK* | 5'UTR;1stExon | Island | -0.949 |
| cg10459387 | 3 | 180319718 | *TTC14;TTC14* | TSS200;TSS200 | Shore | 0.948 |
| cg13133420 | 18 | 21718863 | *CABYR;CABYR;CABYR;CABYR;CABYR;CABYR* | TSS200;TSS200;TSS200;TSS200;TSS1500;TSS200 | Island | -0.948 |
| cg22647996 | 5 | 141393674 | *GNPDA1* | TSS1500 | Shore | -0.948 |

^1^Identification of the CpG according to EPIC array; ^2^Chromosome; ^3^Δβ-values (β-value CTC-MCC-41 - β-value HT29). All CpGs in the table are significant (FDR adjusted p value < 0.05) and are arranged according to their absolute Δβ-value.

**Supplementary Table 7.** Top 10 differentially methylated genes related to biological processes identified in CTC-MCC-41 in comparison to HT29 cells

| **Biological process** | **Top 10 differentially methylated genes^1^** |
| --- | --- |
| Regulation of transcription | ZNF71, MTERF, ZCCHC12, FADS1, RUNX3, HLTF, ZNF773, MEF2C, ZNF419, AIRE |
| Signal transduction | CABYR, ARHGAP4, RASL11B, NTF3, DLC1, PRKACB, FST, TOLLIP, ABR, CDC42SE1 |
| Transmembrane transport | HLTF, GABRA2, SLC7A2, SCN9A, PRKACB, SLC38A3, SCN5A, TRPC3, KCNH2, DST |
| Transport | FADS1, SLC7A2, TMEM9, CYGB, TXNRD1, SLC30A3, GRIN2B, STARD4, GABRA4, UQCRC1 |
| Cell differentiation | ANPEP, BMPR1B, ARX, ZNF423, RBM24, FEZF1, INHA, SFRP5, PAX6, NHLH2 |
| Cell adhesion | CELSR1, C1orf38, CDHR2, DST, PCDH7, IRS1, NID2, PARVB, PLXNC1, LEF1 |
| Apoptotic process | RASSF5, ARHGAP4, TNFRSF19, MEF2C, PNMA1, ABR, BRCA1, TNFRSF10C, FAM176A, BAX |
| Cell cycle | SEPT5, SMPD3, SEPT9, BRCA1, REPIN1, GSPT2, LZTS2, CHFR, C13orf15, PARD6A |
| Blood coagulation | HLTF, NTF3, PRKACB, KLF7, GATA2, TRPC3, PLCG2, FYN, KIF3B, RAPGEF3 |
| Positive regulation of cell proliferation | MATK, FST, CCK, DST, MIAT, HOXA3, IRS1, WWTR1, HSF4, MPST |
| Cell division | SEPT9, LZTS2, CHFR, RCC2, PARD6A, TUBA1A, USP37, BCAR1, CINP, CETN1 |
| Cellular protein metabolic process | KLF7, GSPT2, ST3GAL4, FURIN, ST8SIA2, RPL31, BBS10, TAF7, RPN1, TUBA1A |
| Metabolic process | HLTF, ANPEP, SMPD3, GSTM3, CPE, RIMKLB, NEU1, UCKL1, TGM2, PM20D1 |
| Carbohydrate metabolic process | GNPDA1, PRKACB, B4GALNT1, GALC, ST8SIA2, PFKM, PGAM2, CENPC, KL, IDH2 |
| DNA repair | BRCA1, RPA2, FANCA, MORF4L1, CINP, EME1, TAOK3, LIG4, POLA1, FBXO6 |
| Mitotic cell cycle | RPA2, CHFR, RCC2, TUBA1A, CSNK1E, PKMYT1, TUBA4A, CENPA, PSMA1, PPP2R1A |

^1^In each Biological process, genes are arranged from left to right in decreasing order of absolute Δβ-value between CTC-MCC-41 and HT29 cells.

**Supplementary Table 8.** Top 10 differentially methylated genes related to Panther pathways identified in CTC-MCC-41 in comparison to HT29 cells

| **Panther pathways** | **Top 10 differentially methylated genes^1^** |
| --- | --- |
| Wnt signaling pathway | CELSR1, HLTF, BMPR1B, CDHR2, ANKRD6, PCDH7, SFRP5, LEF1, NFATC1, CTBP1 |
| Cadherin signaling pathway | CELSR1, CDHR2, FYN, PCDH7, LEF1, PCDHAC2, PCDHA1, PCDHA2, PCDHA3, PCDHA4 |
| Inflammation mediated by chemokine and cytokine signaling pathway | PRKACB, PLCG2, PLCL1, NFATC1, PAK1, MAP3K4, ACTA1, SOCS5, PRKCZ, PF4V1 |
| Angiogenesis | PLCG2, PDGFC, PAK1, ARHGAP1, PIK3R1, RBPJL, FRZB, PRKCZ, SOS2, LPXN |
| Integrin signalling pathway | FYN, PARVB, MAP3K4, PIK3R1, BCAR1, LIMS2, SOS2, ITGA7, ITGBL1, PTK2B |
| PDGF signaling pathway | ARHGAP4, DLC1, PLCG2, MAP3K4, RERG, GAB2, PIK3R1, SRF, STAT5A, ARHGAP6 |
| Apoptosis signaling pathway | BAX, ATF1, HSPA2, MADD, EIF2S1, PRKCH, JDP2, MAP3K14, BIK, BCL2L10 |
| TGF-beta signaling pathway | BMP7, BMPR1B, BMP8B, BMP3, ACVR2A, CITED1, BMP2, GDF9, FOXH1, ACVR1B |
| FGF signaling pathway | PLCG2, MAP3K4, FGF22, FGF5, PRKCZ, SOS2, FGF6, PPP2R1A, FGF13, PPP2R1B |
| EGF receptor signaling pathway | PLCG2, PHLDB2, MAP3K4, GAB2, BTC, STAT5A, NF1, PRKCZ, SOS2, MRAS |
| Endothelin signaling pathway | PRKACB, FURIN, EDNRA, PIK3R1, PRKCZ, ADCY9, ADCY4, EDNRB, PRKCH, PRKAR2B |

^1^In each Panther pathway, genes are arranged from left to right in decreasing order of absolute Δβ-value between CTC-MCC-41 and HT29 cells.

**Supplementary Table 9.** Top 50 differentially methylated CpGs identified in CTC-MCC-41 in comparison to COLO205 cells.

| **TargetID^1^** | **Chr^2^** | **Position** | **Gene name** | **Gene region** | **CpG context** | **Δβ^3^** |
| --- | --- | --- | --- | --- | --- | --- |
| cg14052728 | 2 | 95941719 | *PROM2;PROM2;PROM2* | Body;Body;Body |  | 0.981 |
| cg12558358 | 3 | 23244824 | *UBE2E2;UBE2E2* | 1stExon;5'UTR | Island | -0.980 |
| cg15816080 | 7 | 65670215 | *TPST1* | TSS200 | Island | -0.978 |
| cg02478789 | 19 | 9649310 | *ZNF426* | TSS200 | Island | 0.976 |
| cg21028251 | 3 | 133614589 | *RAB6B;RAB6B* | 1stExon;5'UTR | Island | -0.975 |
| cg08935899 | 11 | 6651712 | *DCHS1* | Body | Island | -0.975 |
| cg26472802 | 21 | 45713719 | *AIRE;AIRE* | Body;Body | Island | 0.975 |
| cg15361065 | 3 | 55522301 | *WNT5A* | TSS1500 | Island | 0.974 |
| cg11777419 | 14 | 104604401 | *KIF26A* | TSS1500 | Island | -0.974 |
| cg10975049 | 10 | 72432542 | *ADAMTS14;ADAMTS14* | TSS200;TSS200 | Island | 0.974 |
| cg24091001 | 2 | 172949870 | *DLX1;DLX1* | TSS1500;TSS1500 | Island | -0.973 |
| cg18202521 | 1 | 19600702 | *AKR7L;AKR7L* | TSS200;TSS200 | Island | 0.973 |
| cg21578090 | 17 | 4488018 | *SMTNL2;SMTNL2* | 1stExon;5'UTR | Island | -0.973 |
| cg17192454 | 2 | 74726651 | *LBX2;LBX2* | 1stExon;Body | Island | 0.973 |
| cg04138112 | 1 | 167090618 | *DUSP27* | Body | Island | 0.972 |
| cg09247193 | 17 | 47075095 | *IGF2BP1;IGF2BP1;IGF2BP1;IGF2BP1* | 5'UTR;5'UTR;1stExon;1stExon | Island | -0.971 |
| cg09744766 | 16 | 53407421 |  |  | Island | 0.971 |
| cg12034979 | 12 | 133532882 | *ZNF605;ZNF605;ZNF605* | 1stExon;TSS200;5'UTR | Island | 0.970 |
| cg27047283 | X | 135333567 | *MAP7D3;MAP7D3* | 1stExon;5'UTR | Island | -0.970 |
| cg12087304 | 4 | 122873018 | *TRPC3* | TSS200 | Island | -0.970 |
| cg16112120 | 3 | 133614580 | *RAB6B;RAB6B* | 1stExon;5'UTR | Island | -0.970 |
| cg23755113 | 7 | 65670237 | *TPST1* | TSS200 | Island | -0.970 |
| cg11424828 | 8 | 2075469 | *MYOM2* | Body | Island | 0.970 |

**Supplementary Table 9.** *Continued*

| cg14557185 | 3 | 149374763 | *WWTR1;WWTR1;WWTR1* | Body;Body;Body | Island | 0.970 |
| --- | --- | --- | --- | --- | --- | --- |
| cg24027179 | 17 | 8534165 | *MYH10* | TSS200 | Island | -0.969 |
| cg26802289 | 7 | 20817859 |  |  | Island | 0.969 |
| cg16088539 | 6 | 44044443 |  |  | Shelf | -0.969 |
| cg12798157 | 1 | 19600719 | *AKR7L;AKR7L* | TSS200;TSS200 | Island | 0.969 |
| cg20591405 | 3 | 133614578 | *RAB6B;RAB6B* | 5'UTR;1stExon | Island | -0.968 |
| cg16862295 | 4 | 42399798 | *SHISA3* | TSS200 | Island | -0.967 |
| cg14866200 | 4 | 42399843 | *SHISA3* | TSS200 | Island | -0.966 |
| cg15786128 | 14 | 33408406 | *NPAS3;NPAS3;NPAS3;NPAS3* | TSS200;TSS200;TSS200;TSS200 |  | -0.966 |
| cg08287334 | 19 | 1854633 | *KLF16* | Body | Island | 0.966 |
| cg17104258 | 1 | 167090646 | *DUSP27* | Body | Island | 0.966 |
| cg11376198 | 1 | 19600730 | *AKR7L;AKR7L* | TSS200;TSS200 | Island | 0.966 |
| cg04165935 | 12 | 133532880 | *ZNF605;ZNF605;ZNF605* | 1stExon;TSS200;5'UTR | Island | 0.966 |
| cg00740547 | 21 | 46439236 |  |  | Island | -0.966 |
| cg15616946 | 18 | 11689206 | *GNAL;GNAL* | 5'UTR;1stExon | Island | -0.965 |
| cg22714290 | 1 | 207992548 | *LOC148696* | Body |  | 0.965 |
| cg13633361 | 12 | 127631167 |  |  | Island | -0.965 |
| cg07446674 | X | 135333561 | *MAP7D3;MAP7D3* | 1stExon;5'UTR | Island | -0.965 |
| cg24713878 | 17 | 3599481 | *P2RX5;P2RX5;P2RX5;P2RX5;P2RX5;P2RX5* | 1stExon;1stExon;5'UTR;5'UTR;1stExon;5'UTR | Island | -0.965 |
| cg26022183 | 1 | 156215655 | *PAQR6;PAQR6* | 5'UTR;Body | Island | -0.964 |
| cg09243759 | 13 | 77460613 | *KCTD12* | TSS200 | Island | -0.964 |
| cg02728595 | 3 | 196255632 |  |  | Island | -0.964 |
| cg19083459 | 12 | 28122371 | *PTHLH;PTHLH;PTHLH;PTHLH* | Body;Body;Body;Body | Shore | 0.964 |
| cg18031307 | 2 | 74726649 | *LBX2;LBX2* | 1stExon;Body | Island | 0.964 |

**Supplementary Table 9.** *Continued*

| cg06377278 | 1 | 25256369 | *RUNX3;RUNX3;RUNX3* | 1stExon;Body;5'UTR | Island | -0.963 |
| --- | --- | --- | --- | --- | --- | --- |
| cg22089737 | 2 | 74726633 | *LBX2;LBX2* | 1stExon;Body | Island | 0.963 |
| cg15913725 | 2 | 3286479 | *TSSC1* | Body | Island | 0.963 |

^1^Identification of the CpG according to EPIC array; ^2^Chromosome; ^3^Δβ-values (β-value CTC-MCC-41 - β-value COLO205). All CpGs in the table are significant (FDR adjusted p value < 0.05) and are arranged according to their absolute Δβ-value.

**Supplementary Table 10.** Top 50 differentially methylated CpGs from CpG islands or shore regions of gene promoters identified in CTC-MCC-41 in comparison to COLO205 cells.

| **TargetID^1^** | **Chr^2^** | **Position** | **Gene name** | **Gene region** | **CpG context** | **Δβ^3^** |
| --- | --- | --- | --- | --- | --- | --- |
| cg12558358 | 3 | 23244824 | *UBE2E2;UBE2E2* | 1stExon;5'UTR | Island | -0.980 |
| cg15816080 | 7 | 65670215 | *TPST1* | TSS200 | Island | -0.978 |
| cg02478789 | 19 | 9649310 | *ZNF426* | TSS200 | Island | 0.976 |
| cg21028251 | 3 | 133614589 | *RAB6B;RAB6B* | 1stExon;5'UTR | Island | -0.975 |
| cg15361065 | 3 | 55522301 | *WNT5A* | TSS1500 | Island | 0.974 |
| cg11777419 | 14 | 104604401 | *KIF26A* | TSS1500 | Island | -0.974 |
| cg10975049 | 10 | 72432542 | *ADAMTS14;ADAMTS14* | TSS200;TSS200 | Island | 0.974 |
| cg24091001 | 2 | 172949870 | *DLX1;DLX1* | TSS1500;TSS1500 | Island | -0.973 |
| cg18202521 | 1 | 19600702 | *AKR7L;AKR7L* | TSS200;TSS200 | Island | 0.973 |
| cg21578090 | 17 | 4488018 | *SMTNL2;SMTNL2* | 1stExon;5'UTR | Island | -0.973 |
| cg17192454 | 2 | 74726651 | *LBX2;LBX2* | 1stExon;Body | Island | 0.973 |
| cg09247193 | 17 | 47075095 | *IGF2BP1;IGF2BP1;IGF2BP1;IGF2BP1* | 5'UTR;5'UTR;1stExon;1stExon | Island | -0.971 |
| cg12034979 | 12 | 133532882 | *ZNF605;ZNF605;ZNF605* | 1stExon;TSS200;5'UTR | Island | 0.970 |
| cg27047283 | X | 135333567 | *MAP7D3;MAP7D3* | 1stExon;5'UTR | Island | -0.970 |
| cg12087304 | 4 | 122873018 | *TRPC3* | TSS200 | Island | -0.970 |
| cg16112120 | 3 | 133614580 | *RAB6B;RAB6B* | 1stExon;5'UTR | Island | -0.970 |
| cg23755113 | 7 | 65670237 | *TPST1* | TSS200 | Island | -0.970 |
| cg24027179 | 17 | 8534165 | *MYH10* | TSS200 | Island | -0.969 |
| cg12798157 | 1 | 19600719 | *AKR7L;AKR7L* | TSS200;TSS200 | Island | 0.969 |
| cg20591405 | 3 | 133614578 | *RAB6B;RAB6B* | 5'UTR;1stExon | Island | -0.968 |
| cg16862295 | 4 | 42399798 | *SHISA3* | TSS200 | Island | -0.967 |

**Supplementary Table 10.** *Continued*

| cg14866200 | 4 | 42399843 | *SHISA3* | TSS200 | Island | -0.966 |
| --- | --- | --- | --- | --- | --- | --- |
| cg11376198 | 1 | 19600730 | *AKR7L;AKR7L* | TSS200;TSS200 | Island | 0.966 |
| cg04165935 | 12 | 133532880 | *ZNF605;ZNF605;ZNF605* | 1stExon;TSS200;5'UTR | Island | 0.966 |
| cg15616946 | 18 | 11689206 | *GNAL;GNAL* | 5'UTR;1stExon | Island | -0.965 |
| cg07446674 | X | 135333561 | *MAP7D3;MAP7D3* | 1stExon;5'UTR | Island | -0.965 |
| cg24713878 | 17 | 3599481 | *P2RX5;P2RX5;P2RX5;P2RX5;P2RX5;P2RX5* | 1stExon;1stExon;5'UTR;5'UTR;1stExon;5'UTR | Island | -0.965 |
| cg26022183 | 1 | 156215655 | *PAQR6;PAQR6* | 5'UTR;Body | Island | -0.964 |
| cg09243759 | 13 | 77460613 | *KCTD12* | TSS200 | Island | -0.964 |
| cg18031307 | 2 | 74726649 | *LBX2;LBX2* | 1stExon;Body | Island | 0.964 |
| cg06377278 | 1 | 25256369 | *RUNX3;RUNX3;RUNX3* | 1stExon;Body;5'UTR | Island | -0.963 |
| cg22089737 | 2 | 74726633 | *LBX2;LBX2* | 1stExon;Body | Island | 0.963 |
| cg16684259 | 17 | 33570030 | *SLFN5* | TSS200 | Island | -0.963 |
| cg14551002 | 17 | 31552 | *DOC2B* | TSS200 | Island | -0.962 |
| cg20696912 | 3 | 183146011 | *MCF2L2* | TSS200 | Island | -0.962 |
| cg04210573 | X | 71351784 | *RGAG4;NHSL2* | TSS200;Body | Island | -0.962 |
| cg25249518 | 6 | 119399928 | *FAM184A;FAM184A* | 5'UTR;TSS200 | Island | -0.962 |
| cg10445453 | 10 | 72432540 | *ADAMTS14;ADAMTS14* | TSS200;TSS200 | Island | 0.962 |
| cg11929643 | 3 | 48700391 | *CELSR3* | TSS200 | Island | -0.962 |
| cg19614698 | 14 | 104604893 | *KIF26A* | TSS200 | Island | -0.962 |
| cg25230111 | 19 | 56159817 | *CCDC106* | 5'UTR | Island | 0.961 |
| cg24426788 | X | 48931572 | *PRAF2* | 1stExon | Island | 0.961 |
| cg03899721 | 8 | 8748792 | *MFHAS1* | 1stExon | Island | 0.961 |
| cg09823095 | 7 | 130419042 | *KLF14* | TSS200 | Island | -0.961 |

**Supplementary Table 10.** *Continued*

| cg15653282 | 18 | 11689218 | *GNAL;GNAL* | 5'UTR;1stExon | Island | -0.961 |
| --- | --- | --- | --- | --- | --- | --- |
| cg12728517 | 11 | 6947552 | *ZNF215* | TSS200 | Island | -0.961 |
| cg23965061 | 8 | 22960599 | *TNFRSF10C;TNFRSF10C* | 5'UTR;1stExon | Island | 0.960 |
| cg01565320 | 4 | 42399851 | *SHISA3* | TSS200 | Island | -0.960 |
| cg16630791 | 16 | 86543538 | *FOXF1* | TSS1500 | Island | -0.960 |
| cg24838063 | 12 | 130822603 | *PIWIL1* | TSS200 | Island | 0.960 |

^1^Identification of the CpG according to EPIC array; ^2^Chromosome; ^3^Δβ-values (β-value CTC-MCC-41 - β-value COLO205). All CpGs in the table are significant (FDR adjusted p value < 0.05) and are arranged according to their absolute Δβ-value.

**Supplementary Table 11.** Promoter hypermethylated genes obtained by DNA genome-wide DNA methylation analysis with EPIC array in CTC-MCC-41 respect to HT29 cells selected for validation.

| **TargetID^1^** | **Chr^2^** | **Position** | **Gene name** | **Gene region** | **CpG context** | | **Δβ^3^** |
| --- | --- | --- | --- | --- | --- | --- | --- |
| cg12621514 | 10 | 54073047 | *DKK1* | TSS1500 | | Shore | 0.518 |
| cg25158147 | 10 | 54073150 | *DKK1* | TSS1500 | | Shore | 0.313 |
| cg11988964 | 10 | 54073189 | *DKK1* | TSS1500 | | Shore | 0.377 |
| cg02302582 | 10 | 54073281 | *DKK1* | TSS1500 | | Shore | 0.635 |
| cg11931116 | 10 | 54074040 | *DKK1;DKK1* | 5'UTR;1stExon | | Island | 0.703 |
| cg09445939 | 10 | 54074079 | *DKK1;DKK1* | 5'UTR;1stExon | | Island | 0.576 |
| **cg07684796** | **10** | **54074209** | ***DKK1*** | **1stExon** | | **Island** | **0.750** |
| cg27591349 | 10 | 54074250 | *DKK1* | 1stExon | | Island | 0.67 |
| cg26677741 | 10 | 54074260 | *DKK1;PRKG1-AS1* | 1stExon;TSS1500 | | Island | 0.439 |
| cg02836487 | 3 | 128206457 | *GATA2;GATA2;GATA2* | 5'UTR;5'UTR;5'UTR | | Island | 0.262 |
| cg00847029 | 3 | 128206844 | *GATA2;GATA2;GATA2* | 5'UTR;5'UTR;TSS200 | | Island | 0.436 |
| cg23520930 | 3 | 128206967 | *GATA2;GATA2;GATA2* | 5'UTR;5'UTR;TSS1500 | | Island | 0.452 |
| cg09024124 | 3 | 128207255 | *GATA2;GATA2;GATA2;GATA2* | 1stExon;5'UTR;5'UTR;TSS1500 | | Island | 0.597 |
| **cg02980693** | **3** | **128208970** | ***GATA2*** | **5'UTR** | | **Island** | **0.897** |
| cg13808674 | 3 | 128209319 | *GATA2* | 5'UTR | | Island | 0.750 |
| cg04101969 | 13 | 20806477 | *GJB6;GJB6;GJB6;GJB6;GJB6;GJB6;GJB6* | 5'UTR;1stExon;1stExon;5'UTR;5'UTR;1stExon;TSS1500 | | Island | 0.453 |
| **cg14467488** | **13** | **20806663** | ***GJB6;GJB6;GJB6;GJB6*** | **TSS200;TSS200;TSS200;TSS1500** | | **Island** | **0.305** |
| cg03460558 | X | 128657473 | *SMARCA1;SMARCA1* | TSS200;TSS200 | | Shore | 0.457 |
| **cg08617833** | **X** | **128657697** | ***SMARCA1;SMARCA1*** | **TSS1500;TSS1500** | | **Shore** | **0.294** |
| **cg26010110** | **X** | **128657699** | ***SMARCA1;SMARCA1*** | **TSS1500;TSS1500** | | **Shore** | **0.258** |
| cg20736997 | 4 | 157891938 | *PDGFC* | 1stExon | | Shore | 0.547 |
| cg26777861 | 4 | 157892013 | *PDGFC* | 1stExon | | Shore | 0.796 |
| cg09848659 | 4 | 157892087 | *PDGFC;PDGFC* | 5'UTR;1stExon | | Shore | 0.725 |
| **cg02278887** | **4** | **157892159** | ***PDGFC;PDGFC;PDGFC*** | **1stExon;5'UTR;Body** | | **Shore** | **0.753** |
| **cg03376310** | **4** | **157892187** | ***PDGFC;PDGFC;PDGFC*** | **1stExon;5'UTR;Body** | | **Shore** | **0.758** |
| cg27110994 | 4 | 157892489 | *PDGFC;PDGFC* | 5'UTR;1stExon | | Shore | 0.234 |
| cg24634420 | 4 | 157892748 | *PDGFC* | TSS1500 | | Island | 0.435 |
| cg22451233 | 4 | 157892765 | *PDGFC* | TSS1500 | | Island | 0.220 |
| cg13153190 | 4 | 157893231 | *PDGFC* | TSS1500 | | Island | 0.354 |
| cg04419157 | 4 | 157893306 | *PDGFC* | TSS1500 | | Shore | 0.299 |
| cg07537734 | 4 | 157893541 | *PDGFC* | TSS1500 | | Shore | 0.289 |
| cg25147026 | 1 | 186650441 | *PTGS2* | TSS1500 | | Shore | 0.335 |
| **cg16101346** | **1** | **186650479** | ***PTGS2*** | **TSS1500** | | **Shore** | **0.369** |
| cg14598116 | 1 | 218518317 | *TGFB2;TGFB2;TGFB2-AS1* | TSS1500;TSS1500;Body | | Shore | 0.418 |
| **cg06899755** | **1** | **218520325** | ***TGFB2;TGFB2*** | **1stExon;1stExon** | | **Island** | **0.319** |

^1^Identification of the CpG according to EPIC array; ^2^Chromosome; ^3^Δβ-values (β-value CTC-MCC-41 - β-value HT29). All CpGs in the table are significant (FDR adjusted p value < 0.05) and are arranged according to the alphabetical order of the genes and the genomic position. CpGs selected for validation are indicated in bold.

**Supplementary Table 12.** Promoter hypomethylated genes obtained by genome-wide DNA methylation analysis with EPIC array in CTC-MCC-41 respect to HT29 cells selected for validation.

| **TargetID^1^** | **Chr^2^** | **Position** | **Gene name** | **Gene region** | **CpG context** | **Δβ^3^** |
| --- | --- | --- | --- | --- | --- | --- |
| cg12270822 | 7 | 87229722 | *ABCB1* | 5'UTR | Island | -0.300 |
| cg16772035 | 7 | 87229880 | *ABCB1* | 5'UTR | Island | -0.266 |
| **cg09105881** | **7** | **87230060** | ***ABCB1*** | **5'UTR** | **Island** | **-0.275** |
| cg16288248 | 7 | 87230087 | *ABCB1* | 5'UTR | Island | -0.227 |
| cg04599912 | 7 | 87257030 | *RUNDC3B;RUNDC3B;RUNDC3B;ABCB1* | TSS1500;TSS1500;TSS1500;5'UTR | Island | -0.350 |
| cg04710402 | 7 | 87257074 | *RUNDC3B;RUNDC3B;ABCB1;RUNDC3B* | TSS1500;TSS1500;5'UTR;TSS1500 | Island | -0.283 |
| cg00031165 | 7 | 87258360 | *RUNDC3B;ABCB1;RUNDC3B;RUNDC3B* | Body;5'UTR;Body;Body | Island | -0.535 |
| cg12040846 | 7 | 87258443 | *RUNDC3B;ABCB1;RUNDC3B;RUNDC3B* | Body;5'UTR;Body;Body | Island | -0.256 |
| **cg03874092** | **2** | **60780415** | ***BCL11A;BCL11A;BCL11A;BCL11A;BCL11A;BCL11A*** | **1stExon;1stExon;1stExon;5'UTR;5'UTR;5'UTR** | **Shore** | **-0.490** |
| **cg03754165** | **2** | **60780427** | ***BCL11A;BCL11A;BCL11A;BCL11A;BCL11A;BCL11A*** | **1stExon;1stExon;1stExon;5'UTR;5'UTR;5'UTR** | **Shore** | **-0.526** |
| cg15091323 | 2 | 60780474 | *BCL11A;BCL11A;BCL11A;BCL11A;BCL11A;BCL11A* | 1stExon;1stExon;1stExon;5'UTR;5'UTR;5'UTR | Shore | -0.266 |
| **cg26516759** | **20** | **55840973** | ***BMP7*** | **1stExon** | **Island** | **-0.750** |
| cg20340302 | 20 | 55841149 | *BMP7* | 1stExon | Island | -0.955 |
| cg20292547 | 20 | 55841342 | *BMP7;BMP7* | 1stExon;5'UTR | Island | -0.380 |
| cg11670519 | 20 | 55841506 | *BMP7;BMP7* | 1stExon;5'UTR | Island | -0.451 |
| cg23482891 | 20 | 55841841 | *BMP7* | TSS200 | Shore | -0.782 |
| cg14839404 | 20 | 55841869 | *BMP7* | TSS200 | Shore | -0.358 |
| cg15069906 | 20 | 55841888 | *BMP7* | TSS200 | Shore | -0.596 |
| cg03573446 | 20 | 55841893 | *BMP7* | TSS200 | Shore | -0.555 |
| cg22047703 | 20 | 55841896 | *BMP7* | TSS200 | Shore | -0.578 |
| cg10941747 | 20 | 55841898 | *BMP7* | TSS200 | Shore | -0.695 |
| cg12518442 | 20 | 55841906 | *BMP7* | TSS200 | Shore | -0.730 |
| cg00309582 | 20 | 55841925 | *BMP7* | TSS1500 | Shore | -0.749 |
| cg18759209 | 20 | 55842071 | *BMP7* | TSS1500 | Shore | -0.457 |

**Supplementary Table 12.** *Continued*

| cg14782437 | 12 | 4381731 | *CCND2* | TSS1500 | Island | -0.244 |
| --- | --- | --- | --- | --- | --- | --- |
| **cg25425078** | **12** | **4381777** | ***CCND2*** | **TSS1500** | **Island** | **-0.294** |
| **cg15993083** | **12** | **4381788** | ***CCND2*** | **TSS1500** | **Island** | **-0.347** |
| **cg13575161** | **12** | **4381792** | ***CCND2*** | **TSS1500** | **Island** | **-0.263** |
| **cg10091994** | **12** | **4381803** | ***CCND2*** | **TSS1500** | **Island** | **-0.498** |
| cg22500428 | 12 | 4381882 | *CCND2* | TSS1500 | Island | -0.603 |
| cg05667158 | 12 | 4381890 | *CCND2* | TSS1500 | Island | -0.450 |
| cg19582614 | 12 | 4381894 | *CCND2* | TSS1500 | Island | -0.301 |
| cg08553284 | 12 | 4381997 | *CCND2* | TSS1500 | Island | -0.250 |
| cg00814733 | 12 | 4382051 | *CCND2* | TSS1500 | Island | -0.387 |
| cg17296482 | 12 | 4382188 | *CCND2* | TSS1500 | Island | -0.332 |
| **cg26910092** | **2** | **216300599** | ***FN1;FN1;FN1;FN1;FN1;FN1;FN1;FN1;FN1;FN1;FN1;FN1;FN1;FN1*** | **5'UTR;1stExon;1stExon;5'UTR;1stExon;5'UTR;5'UTR;1stExon;1stExon;5'UTR;5'UTR;5'UTR;1stExon;1stExon** | **Island** | **-0.413** |
| cg14190674 | 2 | 216300738 | *FN1;FN1;FN1;FN1;FN1;FN1;FN1;FN1;FN1;FN1;FN1;FN1;FN1;FN1* | 5'UTR;1stExon;1stExon;5'UTR;1stExon;5'UTR;5'UTR;1stExon;1stExon;5'UTR;5'UTR;5'UTR;1stExon;1stExon | Island | -0.299 |
| cg22511947 | 2 | 216301558 | *FN1;FN1;FN1;FN1;FN1;FN1;FN1* | TSS1500;TSS1500;TSS1500;TSS1500;TSS1500;TSS1500;TSS1500 | Shore | -0.309 |
| **cg21075278** | **11** | **68452087** | ***GAL;GAL*** | **1stExon;5'UTR** | **Island** | **-0.388** |
| **cg00226931** | **11** | **68452097** | ***GAL;GAL*** | **1stExon;5'UTR** | **Island** | **-0.569** |
| cg11582456 | 5 | 115908931 | *SEMA6A* | 5'UTR | Island | -0.282 |
| **cg07100508** | **5** | **115909921** | ***SEMA6A;SEMA6A*** | **1stExon;5'UTR** | **Island** | **-0.406** |

^1^Identification of the CpG according to EPIC array; ^2^Chromosome; ^3^Δβ-values (β-value CTC-MCC-41 - β-value HT29). All CpGs in the table are significant (FDR adjusted p value < 0.05) and are arranged according to the alphabetical order of the genes and the genomic position. CpGs selected for validation are indicated in bold.

**Supplementary Figure 1.** Distribution of the differentially methylated CpGs (DMCpGs) found in CTC-MCC-41 with respect to HT29 and COLO205 according to location in enhancer regions. HypoM, hypomethylated; HyperM, hypermethylated.

**Supplementary Figure 2**. Gene Ontology (GO) analysis with all of the most frequent biological processes and Panther pathways for the 10,000 most differentially methylated CpGs of CTC-MCC-41 compared to HT29 and located at CpG islands and shore regions of gene promoters.

**Supplementary Figure 3**. Gene Ontology (GO) analysis with all of the most frequent biological processes and Panther pathways for the 10,000 most differentially methylated CpGs of CTC-MCC-41 compared to COLO205 and located at CpG islands and shore regions of gene promoters.

**Supplementary Figure 4**. Validation of the methylation status of some promoter CpGs located at CpG islands or shore regions and differentially methylated between CTC-MCC-41 and HT29. All CpGs were analyzed by pyrosequencing except *GAL*, which was analyzed by qMSP. EPIC array data are expressed as % of methylation. *, p value < 0.05 between CTC-MCC-41 and HT29 after analysis by EPIC array; ≠, p value < 0.05 between CTC-MCC-41 and HT29 after analysis by pyrosequencing or qMSP.
